# A dual-electropolymerized poly(Xylenol orange)@poly(Euchrysine) sensing interface for sustainable simultaneous electroanalysis of 5-fluorouracil and empagliflozin: density functional theory mechanistic elucidation

**DOI:** 10.1007/s00604-026-08402-y

**Published:** 2026-10-02

**Authors:** Rania S. Ibrahim, Hanaa M. Abd El-Wadood, Marwa R. El-Zahry, Antonio Frontera, Noha M. Hosny

**Affiliations:** 1 Pharmaceutical Analytical Chemistry Department, Faculty of Pharmacy, Assiut University, Assiut, 71526 Egypt; 2 Pharmaceutical Chemistry Department, Faculty of Pharmacy, Badr University in Assiut, Assiut, 2014101 Egypt; 3 Departament de Química, Universitat de les Illes Balears, Crta de Valldemossa km 7.5, Palma de Mallorca (Baleares), 07122 Spain

**Keywords:** Empagliflozin, 5-Fluorouracil, Square-wave voltammetry, Euchrysine, Xylenol orange, Sustainability evaluation, Computational analyses

## Abstract

A sensitive and sustainable electroanalytical approach for the simultaneous determination of 5-fluorouracil (5-FU) and empagliflozin (EMPA) is desirable for pharmaceutical and bioanalytical investigations, particularly in preliminary studies exploring their concomitant use and potential protective effects. Herein, a square-wave voltammetric (SWV) method was developed using a sequentially fabricated poly-XO@poly-EUC/PGE sensing interface, prepared by successive electropolymerization of euchrysine (EUC) and xylenol orange (XO) on the surface of pencil graphite electrode (PGE). Electrochemical and morphological characterization confirmed successful electrode modification and improved interfacial electron-transfer properties. The sequential dual-polymer architecture produced a more pronounced analytical response than the corresponding individual polymer-modified electrodes, supporting a synergistic contribution of the two polymeric layers. Under optimized conditions, the proposed SWV method exhibited good linearity, precision (RSD ≤ 1.51%), and accuracy (recoveries of 96.21–103.59%), with detection limits of 0.33 and 0.02 µM for 5-FU and EMPA, respectively. The method also provided satisfactory recoveries in spiked human plasma, demonstrating its potential applicability to biological samples. An Environmental, Performance, and Practicality Index (EPPI) score of 78.0 highlighted the favorable sustainability and practical performance of the method. Density functional theory (DFT) calculations were performed using isolated EUC and XO molecular models and suggested favorable analyte–modifier interactions and provided qualitative molecular-level support for the proposed sensing mechanism, while the estimated oxidation pathway indicated a one-electron irreversible process. Overall, the poly-XO@poly-EUC/PGE platform provides a sensitive, selective, practical, and sustainable approach for the simultaneous electrochemical determination of 5-FU and EMPA, with potential utility in pharmaceutical quality control and bioanalytical studies.

**Graphical abstract:**

**Figure:**
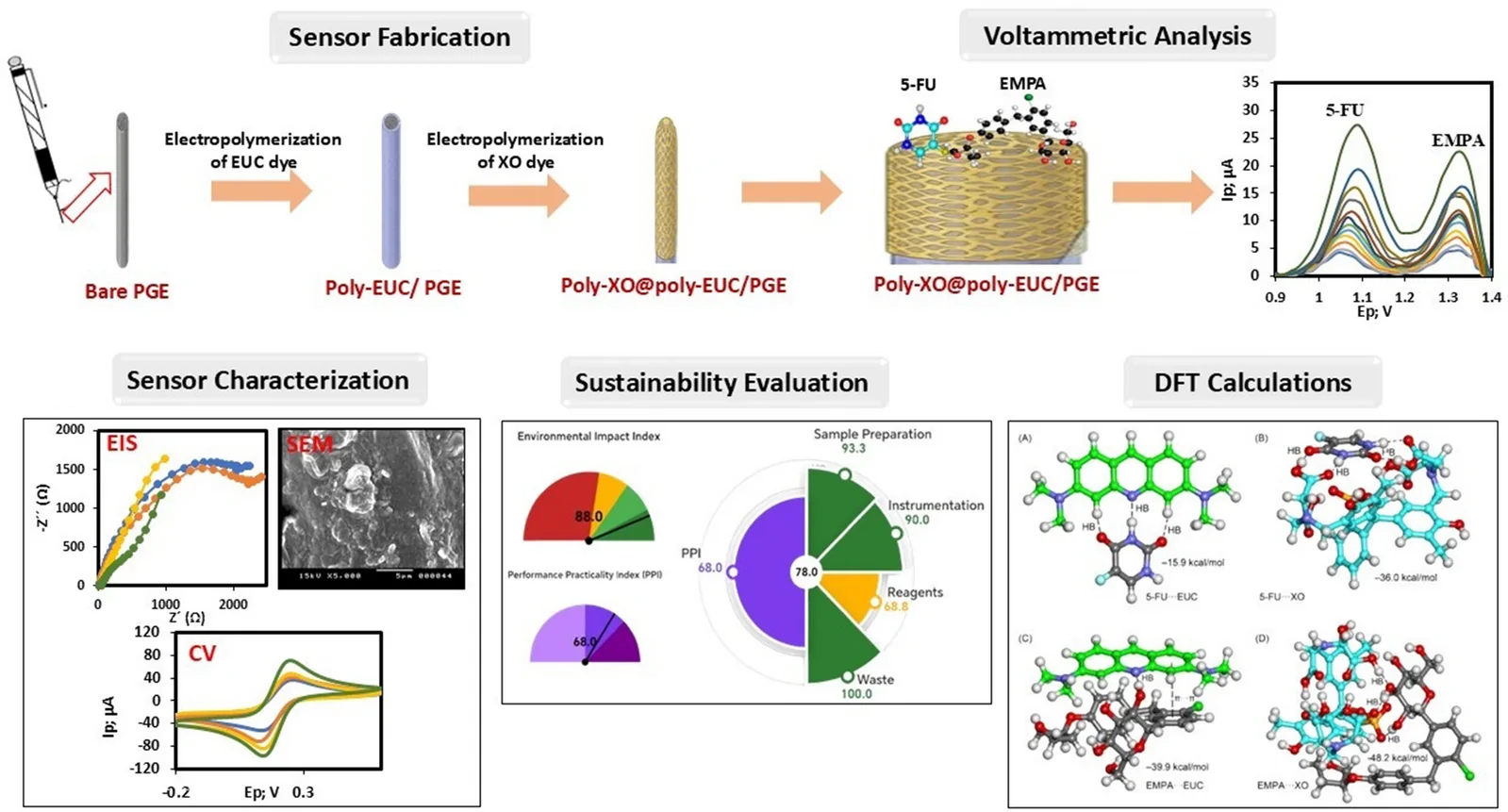

**Supplementary Information:**

The online version contains supplementary material available at https://doi.org/10.1007/s00604-026-08402-y.

## Introduction

Chemotherapy, along with radiation therapy and surgery, represents one of the three principal modalities for cancer treatment. Chemotherapeutic agents are classified into several groups, each characterized by a distinct mechanism of action. These agents exert cytotoxic effects on malignant cells; however, they may also damage normal, rapidly dividing cells, leading to adverse effects that can compromise both survival outcomes and the quality of life of patients receiving antineoplastic therapy. Chemotherapy-induced toxicities vary in severity, ranging from mild effects such as nausea, vomiting, and alopecia to more serious complications, including hematological disorders (anemia, neutropenia, cytopenia, and bone marrow suppression) and organ toxicities affecting vital systems such as the heart, liver, kidneys, and central nervous system [1, 2]. Therefore, minimizing chemotherapy-associated adverse effects is essential for improving both patient survival and quality of life. The co-administration of supportive medications aimed at mitigating these toxicities represents an effective strategy to reduce treatment-related complications [3].

5-Fluorouracil (5-FU) is a chemotherapeutic drug belonging to the antimetabolite category. It is employed in the treatment of various malignancies, including lung, breast, and colorectal cancers. The anticancer activity of 5-FU primarily arises from inhibition of thymidylate synthase enzyme and the integration of its metabolites into DNA and RNA, thereby disrupting nucleic acid synthesis and inducing cell death [4, 5]. However, 5-FU is non-selective and affects both malignant and normal cells, which leads to several adverse effects, including nephrotoxicity and cardiotoxicity [6–8].

Empagliflozin (EMPA) is a gliflozin member of the sodium–glucose cotransporter-2 (SGLT2) inhibitors of antidiabetic agents, commonly used for the management of type 2 diabetes mellitus (T2DM). It reduces blood glucose levels by reducing the renal threshold for glucose and decreasing renal reabsorption of filtered glucose, thereby enhancing urinary glucose excretion. Unlike many antidiabetic drugs, EMPA acts independently of insulin, resulting in a lower risk of hypoglycemia [9, 10]. In addition, EMPA has demonstrated significant nephroprotective and cardioprotective effects, particularly in patients with T2DM and established cardiovascular disease [10, 11]. These protective properties are beneficial in mitigating the cardiotoxicity and nephrotoxicity associated with 5-FU therapy [12, 13]. Elbadr et al. [12] has described the potential protective and therapeutic effects of EMPA in nephrotoxicity induced by 5-FU in rats.The nephroprotective action of EMPA is attributed to its anti-inflammatory and antioxidant effects, as well as regulation of the caspase-3 signaling pathway [12]. Moreover, Refaie et al. [13] has investigated the molecular mechanism of EMPA cardioprotection in 5-FU-induced cardiotoxicity through modulation of SGLT2 and TNFα/TLR/NF-κB signaling pathway in experimental animals. EMPA cardioprotective effects are linked to modulation of SGLT2 activity, which reduces oxidative stress, apoptosis, and inflammation, and suppresses the TNF-α/TLR/NF-κB signaling cascade, an important inflammatory pathway implicated in 5-FU-induced cardiotoxicity [13]. In this context and based on the preliminary studies, the concomitant administration of 5-FU with EMPA is pharmacologically promising for mitigating the toxicity associated with 5-FU therapy.

Several analytical techniques have been informed for the determination of 5-FU and EMPA, either individually or combined with other medications, including spectroscopic [14–19], chromatographic [20–27], and electrochemical methods [28–36]. However, to the best of our knowledge, no analytical procedure has yet been developed for their simultaneous determination. Accordingly, the development of a reliable analytical approach for quantifying this drug mixture is essential to evaluate potential interactions and ensure treatment safety, particularly when sensor-based detection is applied in complex biological matrices.

Compared with conventional chromatographic and spectrophotometric techniques [14–27], electrochemical technique and sensing platforms have attracted considerable interest because of their remarkable analytical performance and broad applicability. The combination of high electrical conductivity, extensive surface area, excellent chemical robustness, biocompatibility, and convenient surface modification of the sensing platforms provides favorable conditions for efficient sensor design. As a result, these platforms offer a powerful approach for the rapid and cost-effective determination of diverse analytes, while maintaining high levels of sensitivity and selectivity in a wide range of analytical and biomedical applications [37–44].

Pencil graphite electrodes (PGEs), among the most widely used electrochemical substrates, provide several advantages, including disposability, low cost, ease of modification, environmental friendliness, and excellent sensitivity, making them ideal for the determination of various analytes [37–39]. The surface of PGEs can be readily modified with different materials, such as electropolymerized dyes, to enhance sensitivity and selectivity for pharmaceutical analysis [37–39, 45]. In the present work, a novel sensing platform, poly-XO@poly-EUC/PGE, is introduced for the simultaneous separation and determination of 5-FU and EMPA in both pure solutions and complex biological matrices, including human plasma. The sensor is fabricated by sequential electropolymerization of euchrysine (EUC) followed by xylenol orange (XO) onto the PGE surface.

Euchrysine (EUC), known as acridine orange, is a heterocyclic organic fluorescent redox-active dye that exhibits several biological properties, including antimicrobial, antifungal, and antibacterial activities. It also acts as a nucleic acid marker through intercalation with DNA and RNA [46]. Polymerized EUC-modified electrodes have been widely explored for electrochemical sensing, where EUC demonstrates distinct electrocatalytic properties that enable the establishment of sensitive and practical methodologies for detecting various analytes in biological and pharmaceutical samples [38, 47–52].

Xylenol orange (XO) is an electroactive polybasic (hexabasic) dye belonging to the triphenylmethane family [53, 54]. After electropolymerization, XO-modified electrodes have demonstrated significant potential for the sensitive and reproducible determination of numerous analytes [53–58].

Accordingly, a newly designed sensor based on sequentially electropolymerized XO layers over polymerized EUC layers (poly-XO@poly-EUC/PGE) is proposed for the concurrent separation and determination of 5-FU and EMPA in both pure form and human plasma samples. The electrochemical performance of the sensing platform was examined using scanning electron microscopy (SEM), cyclic voltammetry (CV), and electrochemical impedance spectroscopy (EIS). Based on the fabricated sensor, square wave voltammetric (SWV) methodology was established for concurrent determination of 5-FU-EMPA mixture. The SWV approach is a highly sensitive pulse voltammetric technique that provides an efficient discrimination of faradaic currents from the capacitive/background current, thereby improving the signal-to-background ratio. This is particularly advantageous for the simultaneous determination of 5-FU and EMPA, whose electrochemical responses need to be resolved within the same measurement. In addition, SWV offers rapid analysis, low analyte consumption, and good sensitivity, making it suitable for quantitative trace-level determination. The modified poly-XO@poly-EUC/PGE sensor also provides enhanced electrochemical responses, while the potential separation between the analytical signals enables simultaneous detection of the two analytes. The proposed SWV approach was fully optimized then validated following ICH [59] and EMA [60] guidelines. Computational calculations were performed to reveal the mechanistic interactions of the sensors and oxidation mechanisms of the target analytes. Computational calculations using the conformer-rotamer ensemble sampling tool (CREST) and density functional theory (DFT) were performed to reveal the mechanistic interactions of the sensors and oxidation mechanisms of the target analytes. These quantum chemical simulations elucidate how the host-guest binding energies, spatial frontier molecular orbital distributions, and open-shell spin densities of the generated cation radicals synergistically dictate the enhanced sensitivity and distinct oxidation pathways of the drugs at the dual-polymer interface. On the other hand, the sustainability of the developed SWV method was also assessed through Environmental, Performance, and Practicality Index (EPPI).

## Experimental

### Materials and reagents

5-Fluorouracil (5-FU, 99% purity) was obtained from Hikma Specialized Pharmaceuticals (Cairo, Egypt), while empagliflozin (EMPA, 99% purity) was kindly provided by Global Napi Company (Cairo, Egypt). Euchrysine dye (EUC) was purchased from Fluka AG (Germany) and prepared as a 1.0 mM solution in Milli-Q water. Xylenol orange (XO, 0.0001 M), obtained from Euromedex (France), was prepared by dissolving 0.0034 g of XO in a 50 mL methanolic solution of 0.1 M sodium hydroxide. Methanol was of HPLC grade (Merck, Germany). All chemicals used for preparing phosphate and Britton–Robinson (BR) buffer solutions were obtained from El-Nasr Pharmaceutical Chemicals Company (ADWIC, Egypt). Phosphate buffer solutions (0.1 M) were prepared using mono- and disodium phosphate salts (98% purity). Britton–Robinson buffer solutions (0.04 M, pH 3.0–9.0) were prepared using boric acid (98% purity), phosphoric acid (85% purity), and glacial acetic acid (96% purity), with pH adjustment carried out using 0.2 M NaOH. Potassium chloride, potassium ferrocyanide, and potassium ferricyanide were also supplied by El-Nasr Pharmaceutical Chemicals Co. (Cairo, Egypt). Rotring (HB) pencil graphite bars (6.0 cm × 0.05 cm) were purchased from a local Egyptian supplier and used as working electrodes. Milli-Q water (Milli-Q Eq. 7016, Millipore SAS, France) was employed for the preparation of all solutions. Other chemicals and solvents of analytical grade were utilized in selectivity studies. Drug-free human plasma specimens were offered by the Blood Bank (Assiut University, Assiut, Egypt). This research does not include any direct interaction or involvement with human participants.

### Instruments

Electrochemical scans were carried out on a Gamry Interface 1010E potentiostat (Gamry Instruments, Warminster, PA, USA) in a conservative three-electrode configuration. Bare and modified PGEs were employed as the working electrodes, while an Ag/AgCl (3.0 M KCl) reference probe and an auxiliary electrode of platinum wire. The surface morphology of the unmodified and modified PGEs was characterized using a scanning electron microscope (SEM; JEOL-JSM-5400LV, JEOL Ltd., USA). Various devices included a pH meter (Hanna Instruments, São Paulo, Brazil), an ultrasonic cleaner (Cole-Parmer, Chicago, USA), an analytical balance (AS 220.B, Radwag, Poland), and a laboratory centrifuge operating at 4000 rpm (Bremsen ECCO, Germany).

### Preparation of stock solution of 5-FU and EMPA mixture

A mixed stock solution containing 0.002 M 5-FU and 0.001 M EMPA was prepared by accurately weighing appropriate amounts of both drugs into a 25 mL volumetric flask. About 5 mL of methanol was added, and the mixture was sonicated for 5 min to guarantee complete dissolution. The solution was then diluted to volume with Britton–Robinson buffer (pH 8.0). Working standard solutions of the investigated (5-FU/EMPA) mixture were subsequently prepared by appropriate dilution of the stock mixture by Britton–Robinson buffer (pH 8.0).

### Fabrication of poly-XO@poly-EUC/PGE

To fabricate the poly-XO@poly-EUC/PGE sensor, Rotring (HB) pencil graphite rods were first thoroughly rinsed with Milli-Q water. The graphite bar was mounted upright in the electrochemical cell, with 1.5 cm of its length exposed to and immersed in the test solution. The modification of the PGE involved two successive electropolymerization steps for the deposition of EUC and XO dyes onto the graphite surface.

In the first step, electropolymerization of EUC was performed by immersing the cleaned PGE in an electrochemical unit holding 1.5 mL of EUC solution (0.001 M) mixed with 3.5 mL of phosphate buffer (0.1 M, pH 6.0). Cyclic voltammetry (CV) was carried out for three consecutive cycles over a potential range of − 0.6 - +1.2 V at 0.1 V s⁻¹. The resulting modified probe was denoted as poly-EUC/PGE. The electrode was then mildly rinsed with Milli-Q water [38, 49].

In the second step, the poly-EUC/PGE was immersed in another electrochemical cell containing 1.0 mL of XO solution (0.0001 M) and 4.0 mL of phosphate buffer (0.1 M, pH 5.0). Electropolymerization of XO was achieved by performing five consecutive CV cycles over a potential range of − 0.6 - +2.0 V at 0.1 V s⁻¹ scan rate [54]. This process resulted in the deposition of XO polymer layers onto the poly-EUC/PGE surface, yielding the final modified electrode, denoted as poly-XO@poly-EUC/PGE. Prior to electrochemical measurements, the fabricated sensor was gently rinsed with Milli-Q water. The prepared electrode was then entirely characterized by CV, electrochemical impedance spectroscopy (EIS), and SEM, and subsequently applied for SWV determination of the 5-FU and EMPA combination in plasma samples.

### Electroanalytical procedure for concurrent determination of 5-FU and EMPA mixture

The electropolymerization variables of EUC and XO dyes, the pH of the supporting electrolyte, and the experimental parameters of the SWV method, such as frequency, step potential, in addition to pulse height, were systematically optimized. The optimized SWV procedure was subsequently validated in accordance with EMA [60] and ICH [59] guidelines. Prior to electrochemical measurements, the fabricated poly-XO@poly-EUC/PGE sensor was gently rinsed with Milli-Q water. Each electrochemical measurement was performed using a fresh pencil graphite rod surface under the optimized conditions.

For analysis, an aliquot of either standard or sample solution containing both 5-FU and EMPA was introduced into the electrochemical unit containing 4 mL of 0.04 M Britton–Robinson buffer (pH 8.0), which served as the supporting electrolyte. SWV measurements were conducted using the poly-XO@poly-EUC/PGE sensor under the optimized conditions: frequency of 12 Hz, pulse amplitude of 10 mV, and step potential of 7 mV. Additionally, CV studies were performed to investigate the electrochemical behavior of the studied mixture at a scan rate of 0.1 V s⁻¹ employing both bare and modified PGEs.

### Simultaneous determination of 5-FU and EMPA in human plasma

Blank (drug-free) human plasma models were utilized for the concurrent determination of 5-FU-EMPA mixture by the developed SWV method. The determination of the studied analyte mixture in human plasma was carried out using the fabricated poly-XO@poly-EUC/PGE sensor in conjunction with the proposed SWV procedure. On the day of analysis, icy plasma samples were thawed at room temperature. Aliquots of 1.0 mL of blank plasma were relocated into a series of Eppendorf tubes, followed by the addition of appropriate volumes of the 5-FU and EMPA mixture.

To precipitate plasma proteins and remove potential interfering substances, the volume in each tube was adjusted to 2.0 mL using acetonitrile. The mixtures were vortexed for 1 min and afterward centrifuged at 10,000 rpm for 30 min to ensure complete protein separation. After centrifugation, suitable aliquots of the clear supernatant were collected and moved into an electrochemical unit comprising 4.0 mL of Britton–Robinson buffer of pH 8.0, where analysis was performed using the poly-XO@poly-EUC/PGE sensor.

The final concentrations of the prepared solutions ranged from 1.5 to 100.0 µM for 5-FU and 0.25–50.0 µM for EMPA, which fall within the linear range of the developed SWV method. The reported maximum plasma concentration of 5-FU is 426.20 µM following an intravenous bolus dose of 400 mg m⁻² [61], whereas EMPA reaches a maximum plasma concentration of 0.505 µM after oral administration of a 25 mg dose [62]. SWV measurements were performed under the optimized conditions described in Sect. Electroanalytical procedure for concurrent determination of 5-FU and EMPA mixture. In addition, blank experiments were conducted using unspiked plasma samples under identical conditions to confirm the absence of interfering signals from endogenous matrix components.

### Computational methods

Initially, the CREST (Conformer–Rotamer Ensemble Sampling Tool) was used to explore the most favored orientations for the interaction of 5-FU and EMPA with EUC and XO. This tool was established as a program for conformational sampling at the extended tight-binding level GFN-xTB [63]. The geometries of the global minima were further optimized using DFT methods as detailed below.

Geometry optimizations 5-FU and EMPA complexes with EUC and XO molecules used as models of the sensing interface were carried out using the Turbomole 7.9 software package [64]. These optimizations were performed without symmetry constraints using the BP86 generalized gradient approximation functional, which couples Becke 1988 exchange with Perdew 1986 correlation [65, 66], supplemented with the D4 Grimme dispersion correction [67]. The def2-TZVP triple zeta valence polarized basis set was employed for all atoms [68]. The molecular orbitals and spin density plots were evaluated at the same theoretical level, utilizing the GaussView 6.0 graphical interface program [69] for generating the plots.

### Sustainability evaluation

The Environmental, Performance, and Practicality Index (EPPI), the recent complete framework assessment tool, was employed to evaluate the environmental, performance, and practicality of the anticipated SWV approach [70]. This tool is a combined index system for the Performance and Practicality Index (abbreviated as PPI) and the Environmental Index (abbreviated as EI). By concurrently assessing ecological sustainability, analytical method performance, and practicality, these two indexes offer a thorough evaluation of analytical techniques. A thorough review of environmental impact, analytical performance, and practical viability is made possible by this evaluation tool, which includes all the phases of the assessed analytical process. The EPPI framework assessment tool assigns a predetermined score to each parameter. The final results are expressed in numerical values ranging from 1 to 100 and presented as a pictograph with two sections: the purple section, which represents the analytical method’s performance and practicality with varying degrees of purple, and the green section, which evaluates the method’s greenness from the perspectives of sample preparation, instrumentation, reagents, and waste handling. Each index has a final score of 100 and can be broken down into multiple areas to assess the method’s effectiveness, usefulness, and environmental impact.

## Results and discussion

This study represents the first electrochemical approach for the separation and simultaneous determination of 5-FU and EMPA in plasma models. The method relies on a novel disposable poly-XO@poly-EUC/PGE probe, characterized by high effective surface area, superior electrical conductivity, enhanced electron transfer kinetics, and simple assembly.

### Fabrication of poly-XO@poly-EUC/PGE probe

To construct the proposed poly-XO@poly-EUC/PGE sensor, a sequential two-step electropolymerization process was employed. In the first stage, the EUC dye was deposited onto the surface of a bare PGE, followed by the electropolymerization of a second layer of XO stain. Both stages were performed using multiple CV scans, with each parameter carefully optimized to achieve maximum sensitivity.

**Fig. 1:**
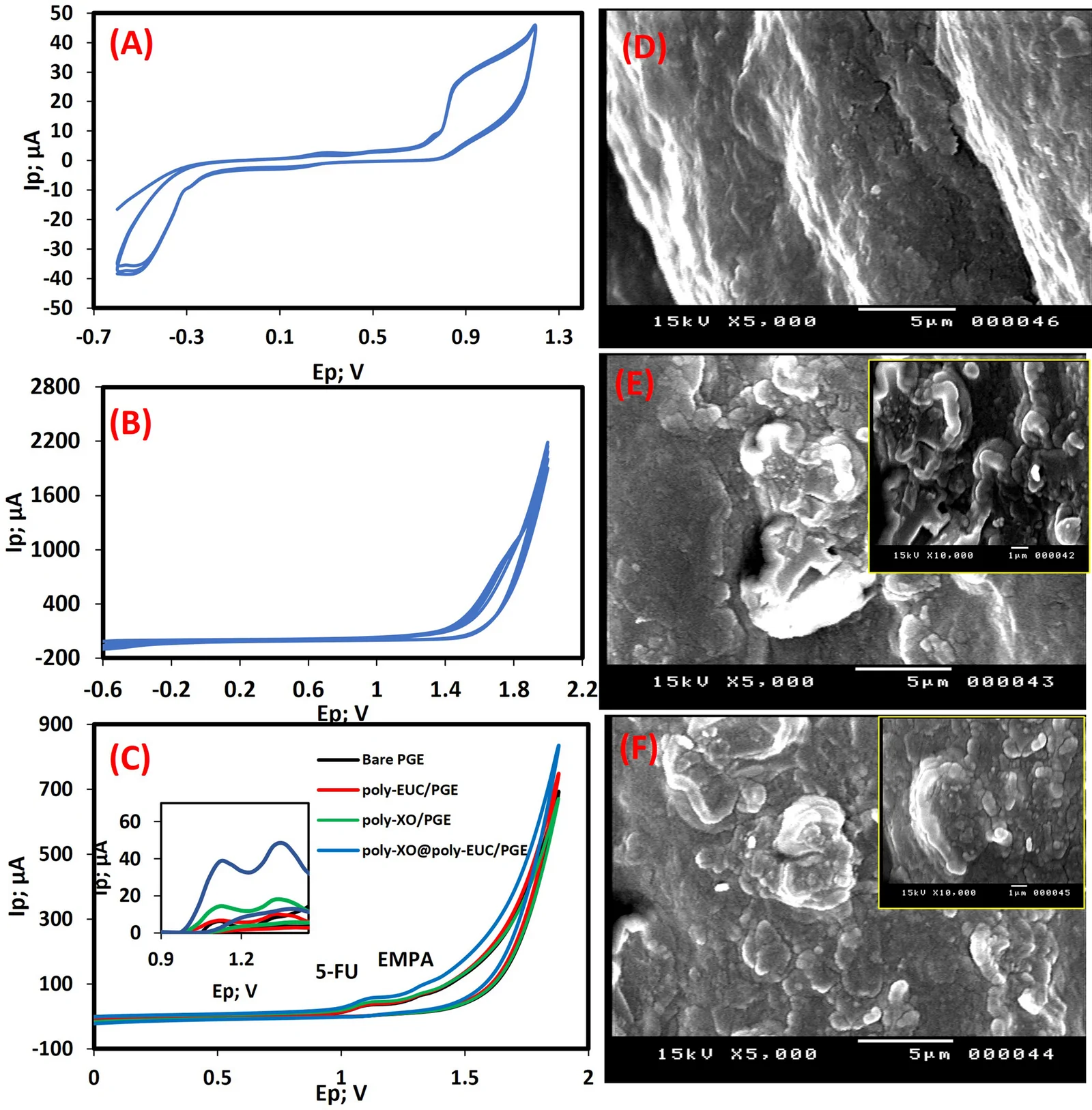
Panel (**A**) Multiple cyclic voltammograms for the electro-polymerization of EUC dye (3 × 10^− 4^ M) in phosphate buffer (0.1 M, pH 6.0) for 3 cycles at 0.1 V s^− 1^ scan rate, (**B**) Multiple cyclic voltammograms for the electro-polymerization of XO dye (2 × 10^− 5^ M) in phosphate buffer (0.1 M, pH 5.0) for 5 cycles at 0.1 V s^− 1^ scan rate, (**C**) Cyclic voltammograms of 1.05 × 10^− 4^ M, and 5.3 × 10^− 5^ M of 5-FU and EMPA, respectively, in Britton-Robinson buffer solution (0.04 M, pH 8.0) recorded at unmodified and modified electrodes, Inset for their corresponding subtracted voltammograms at scan rate 0.1 V s^− 1^. SEM image of bare PGE (**D**), poly-EUC/PGE (**E**), and poly-XO@poly-EUC/PGE (**F**)

First, the optimization parameters for EUC deposition on the bare PGE surface included the volume of EUC, the number of electropolymerization cycles, and the the supporting electrolyte pH. To investigate the effect of EUC volume, different volumes (0.5–2.5 mL) of 0.001 M EUC were examined with respect to the peak current intensities (Ip) of the 5-FU and EMPA mixture. The current responses of both analytes increased as the EUC volume increased from 0.5 to 1.5 mL, achieving extreme values at 1.5 mL. Further increases in the dye volume up to 2.5 mL resulted in a decrease followed by stabilization of the current intensities. Accordingly, 1.5 mL of 0.001 M EUC was selected for subsequent experiments (Fig. 1S, A and B).

The number of electropolymerization cycles also significantly influenced the peak responses of the target analytes. Therefore, cycles ranging from 3 to 15 were investigated. The highest peak currents for both 5-FU and EMPA were obtained using three cycles, after which the responses decreased with increasing cycle number (≥ 5 cycles), likely due to excessive film thickness. Consequently, three electropolymerization cycles were chosen for further studies (Fig. 1S, C and D).

Additionally, the effect of different pHs (3.0–7.0) of phosphate buffer solutions on the EUC electropolymerization and the current responses of 5-FU and EMPA was evaluated. This range covers the acidic-to-near-neutral while avoiding excessively acidic or alkaline media that could adversely affect the stability or electrochemical behavior of the sensing interface. The peak currents increased with increasing pH, reaching maximum values at pH 6.0, and then decreased at pH 7.0. pH 6.0 gave stable dual polymeric layer, resulting in the highest and reproducible current intensities for the investigated analytes. Thus, phosphate buffer at pH 6.0 was selected as the optimal supporting electrolyte for subsequent experiments (Fig. 1S, E and F). The multiple CV curves of EUC under the optimized conditions are presented in Fig. 1A, and the proposed electrochemical oxidation mechanism of the EUC dye has been reported previously [38, 71].

Secondly, the integration of the XO polymeric layer onto the poly-EUC/PGE surface represented the second step in constructing the modified electrode. This stage was influenced by several factors, including dye volume, number of electropolymerization cycles, and pH of the supporting electrolyte.

**Fig. 2:**
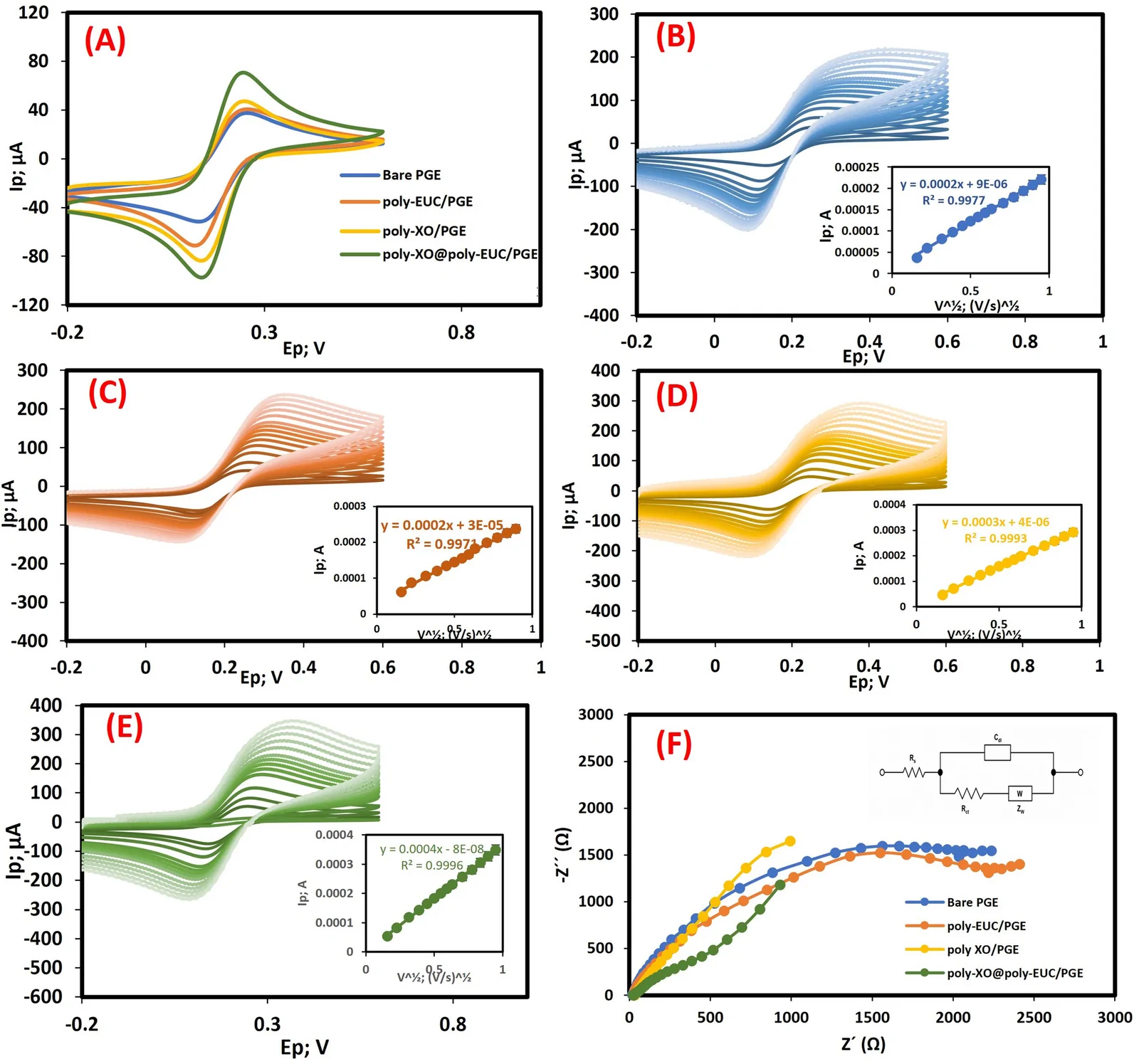
Cyclic voltammograms (**A–E**) and Nyquist curve (**F**) of 1.0 mM of [Fe(CN)_6_]^−3 /−4^ mixture solution in 0.5 M KCl conducted on bare PGE, poly-EUC/PGE, poly-XO/PGE, and poly-XO@poly-EUC/PGE sensor over a potential of -0.2 to + 0.6 V and scan rate of 0.025 V s^− 1^ for (**A**) and 0.025–0.9 V s^− 1^ for (**B**–**E**). Insets of (**B**–**E**) are the linear relationships between the oxidation peak currents and the square root of scan rate. Inset of (**F**) is the Randles equivalent circuit where, R_s_: the electrolyte resistance, C_dl_: double-layer capacitance, R_ct_: the charge transfer resistance, and Z_w_: the Warburg impedance

Diverse volumes (0.5–2.5 mL) of 0.0001 M XO solution were investigated to evaluate their effect on the peak current intensities (Ip) of the 5-FU and EMPA mixture. The Ip values of both analytes increased with increasing XO volume, reaching maximum responses at 1.0 mL. Further increases in dye volume up to 2.5 mL resulted in a gradual decline in current responses, likely due to excessive film thickness. Therefore, 1.0 mL of XO was selected as the ideal volume (Fig. 2S, A and B).

The effect of the number of electropolymerization cycles was also studied over the range of 3–15 cycles. Five cycles provided the best peak separation and highest current responses for both 5-FU and EMPA, whereas further increases in cycle number led to decreased responses. Accordingly, five cycles were selected as the optimum (Fig. 2S, C and D).

Furthermore, the impact of the pH of the phosphate buffer solution used as the supporting electrolyte for XO electropolymerization was estimated within the pH range of 3.0–7.0, avoiding excessively acidic or alkaline media that could adversely affect the stability or electrochemical behavior of the sensing interface. The current intensities diminished when the pH increased from 3.0 to 4.0, followed by a significant increase at pH 5.0, which yielded the best responses for both analytes. Upon further increasing the pH to 6.0, the Ip of 5-FU remained nearly constant and then decreased at pH 7.0, while the Ip of EMPA progressively decreased with increasing pH up to 7.0. Accordingly, pH 5.0 was nominated as the optimal value for subsequent experimentations (Fig. 2S, E and F). Figure 1B illustrates the multiple cyclic voltammograms of XO under the optimized conditions, while the proposed electrochemical oxidation pathway of the XO dye has been reported previously [54].

### Characterization of poly-XO@poly-EUC/PGE

#### SEM examinations

In contrast to the previously examined sensors, including the bare PGE and poly-EUC/PGE, the newly constructed poly-XO@poly-EUC/PGE sensor exhibited superior performance for the concurrent determination of 5-FU and EMPA. Accordingly, the surface morphology of the unmodified PGE, poly-EUC/PGE, and poly-XO@poly-EUC/PGE was investigated using SEM. As displayed in Fig. 1D, the unmodified PGE exhibited a clean and relatively smooth exterior. After electropolymerization of EUC, the SEM image of poly-EUC/PGE (Fig. 1E) revealed the appearance of strip-like structures on the electrode surface, with the insert providing a nearer view of the deposited poly-EUC layer. Further modification with XO dye (Fig. 1F) showed a markedly rougher surface morphology, where the polymerization of XO formed swollen, aggregated structures over the poly-EUC/PGE interface. The inset highlights the poly-XO layer covering the electrode surface. This distinctive rough and porous morphology of the poly-XO@poly-EUC/PGE significantly increases the electroactive surface area, thereby enhancing the sensitivity and enabling improved simultaneous determination of 5-FU and EMPA.

#### Electrochemical performance of the redox system at poly-XO@poly-EUC/PGE by CV and EIS

To explore the electrochemical behavior of the designated poly-XO@poly-EUC/PGE sensor, CV was employed. The electrochemical responses of bare PGE, poly-EUC/PGE, poly-XO/PGE, and poly-XO@poly-EUC/PGE were recorded using 1.0 mM [Fe(CN)_6_]^3−/4−^ as a redox probe in 0.5 M KCl solution, as shown in Fig. 2A–E.

The enhanced anodic and cathodic peak currents observed for the poly-XO@poly-EUC/PGE sensor clearly indicate improved electrochemical performance of the modified sensor. This enhancement is attributed to the augmented electroactive surface area and improved conductivity of the composite polymeric layers compared with poly-EUC/PGE, poly-XO/PGE, as well as the bare PGE.

As illustrated in Fig. 2A, the peak-to-peak separation (ΔE) for the poly-XO@poly-EUC/PGE probe (ΔE = 0.090 V) was significantly lower than that of unmodified PGE (ΔE = 0.161 V), poly-EUC/PGE (ΔE = 0.123 V), and poly-XO/PGE (ΔE = 0.097 V), signifying faster electron transfer kinetics at the recently developed sensing interface.

The improved electrochemical response of the poly-XO@poly-EUC/PGE probe is primarily attributed to its larger electroactive surface area and accelerated electron transfer rate. This was further confirmed by estimating the electroactive surface areas of bare PGE, poly-EUC/PGE, poly-XO/PGE, and poly-XO@poly-EUC/PGE using the Randles–Ševčík equation [72], which is given as follows:

$$I_p=\;(2.69\;\times10^5)\;n^{3/2}\;D_{R}^{1/2}\;\nu^{1/2}\;A_{eff}\;C_0$$

In this context, *C*_*0*_ refers to the [Fe(CN)_6_]^3−/4−^ redox system concentration (1.0 mM), 𝑛 denotes the number of transferred electrons in the redox process, 𝐷_𝑅_ represents the diffusion coefficient (7.6 × 10 ^− 6^ cm^2^ s ^−1^), and 𝜈 corresponds to the scan rate (V s ^− 1^).

To estimate the effective surface area, multiple CV measurements of the [Fe(CN)_6_]^3−/4−^ redox system were recorded over different scan rates ranged from 0.025 to 0.9 V s^− 1^. The square root of the scan rate (ν ^1/2^) was then plotted against the peak current (*I*𝑝), and the slope of the resulting linear plots (insets of Fig. 2B–E) was used for calculation. The obtained slopes were 0.0002 for both bare PGE and poly-EUC/PGE, 0.0003 for poly-XO/PGE, and 0.0004 for poly-XO@poly-EUC/PGE. Accordingly, the calculated electroactive surface areas were 26.95 mm^2^ for both bare PGE and poly-EUC/PGE, 40.43 mm^2^ for poly-XO/PGE, and 53.90 mm^2^ for poly-XO@poly-EUC/PGE, respectively.

Another approach to evaluate the electrochemical performance of the modified electrode was electrochemical impedance spectroscopy (EIS) utilizing the same [Fe(CN)_6_]^3−/4−^ system. Measurements were done over a frequency range of 1.0 Hz to 10 kHz with a perturbation amplitude of 0.01 V, and the Nyquist plots were constructed by correlating the real impedance (Z′) with the imaginary impedance (− Z″). As shown in Fig. 2F (with an enlarged inset of the low-frequency region), both bare PGE and poly-EUC/PGE exhibited pronounced semicircular features, while the poly-XO@poly-EUC/PGE electrode displayed a significantly smaller semicircle diameter, indicating enhanced electron transfer kinetics and improved electrical conductivity. The EIS data were fitted using an equivalent circuit comprising the solution resistance (Rₛ) in series with a parallel network of a constant phase element and a series combination of the charge-transfer resistance (R_ct_) and Warburg impedance (W), see inset of Fig. 2F.

This observation is further supported by the charge transfer resistance (R_ct_) values obtained from EIS fitting, which were 2359.1 Ω for bare PGE, 2322.5 Ω for poly-EUC/PGE, 1179.94 Ω for poly-XO/PGE, and 739.8 Ω for poly-XO@poly-EUC/PGE. These results clearly demonstrate the markedly reduced interfacial resistance and enhanced electron transfer efficiency of the newly developed sensor.

Furthermore, the standard exchange current (I_o_) was determined by the subsequent equation [45], which relates I_o_ to the rate of the electrochemical reaction:

$$I_o=\;RT\;/\;nFR_{ct}$$

Values of 8.314 J.mol⁻¹.K⁻¹, 298 K, 1 electron, and 96,485 C.mol⁻¹ stand for the universal gas constant (R), absolute temperature (T), number of electrons transported (n), and Faraday constant (F), respectively. The calculated I_o_ values for the bare PGE, poly-EUC/PGE, poly-XO/PGE, and poly-XO@poly-EUC/PGE were 10.88, 11.06, 21.76, and 34.71 µA, separately. Notably, the significantly higher I_o_ value found for the poly-XO@poly-EUC/PGE sensor confirms its superior electrocatalytic activity and faster electron-transfer kinetics compared with the other tested electrodes.

### Electrochemical oxidation of 5-FU and EMPA

#### Cyclic voltammetric studies

Using cyclic voltammetry, the electrochemical oxidation behavior of 5-FU and EMPA was inspected at the bare PGE, poly-EUC/PGE, poly-XO/PGE, and the assembled poly-XO@poly-EUC/PGE. As shown in Fig. 1C, the studied mixture exhibited two well-defined anodic peaks corresponding to each drug, where the oxidation peak of 5-FU appeared at + 1.10 V and that of EMPA at + 1.36 V versus Ag/AgCl. No corresponding cathodic peaks were observed, indicating an irreversible electrochemical oxidation process for both analytes.

The unmodified PGE exhibited relatively low current responses for both drugs, confirming its limited electrochemical activity and the necessity of surface modification for analytical applications. Alteration of the probe with poly-EUC resulted in only a marginal enhancement in the current responses of 5-FU and EMPA. The lack of current enhancement with the poly-EUC modified electrode is mainly due to poor film formation, the polymerization is less efficient and weak electrocatalytic activity at pH 8.0, which limits electron transfer between the drugs and the electrode exterior and leading to a thinner, less conductive film with fewer active sites for electron transfer. Additionally, EUC dye is a DNA intercalator and cationic dye. At higher pH, its protonation decreases, reducing its ability to mediate electron transfer and electrostatic attraction. This results in lower current response for the studied analytes [47, 51]. However, further modification through the electropolymerization of XO over the poly-EUC/PGE surface led to a pronounced increase in peak current intensities and significantly improved sensitivity for both analytes. Notably, the poly-XO@poly-EUC/PGE sensor demonstrated superior performance compared to poly-XO/PGE alone, highlighting the synergistic effect of the dual-polymer film in enhancing the electrochemical oxidation signals of 5-FU and EMPA.

#### Effect of scan rate and the expected oxidation pathway of 5-FU and EMPA

**Fig. 3:**
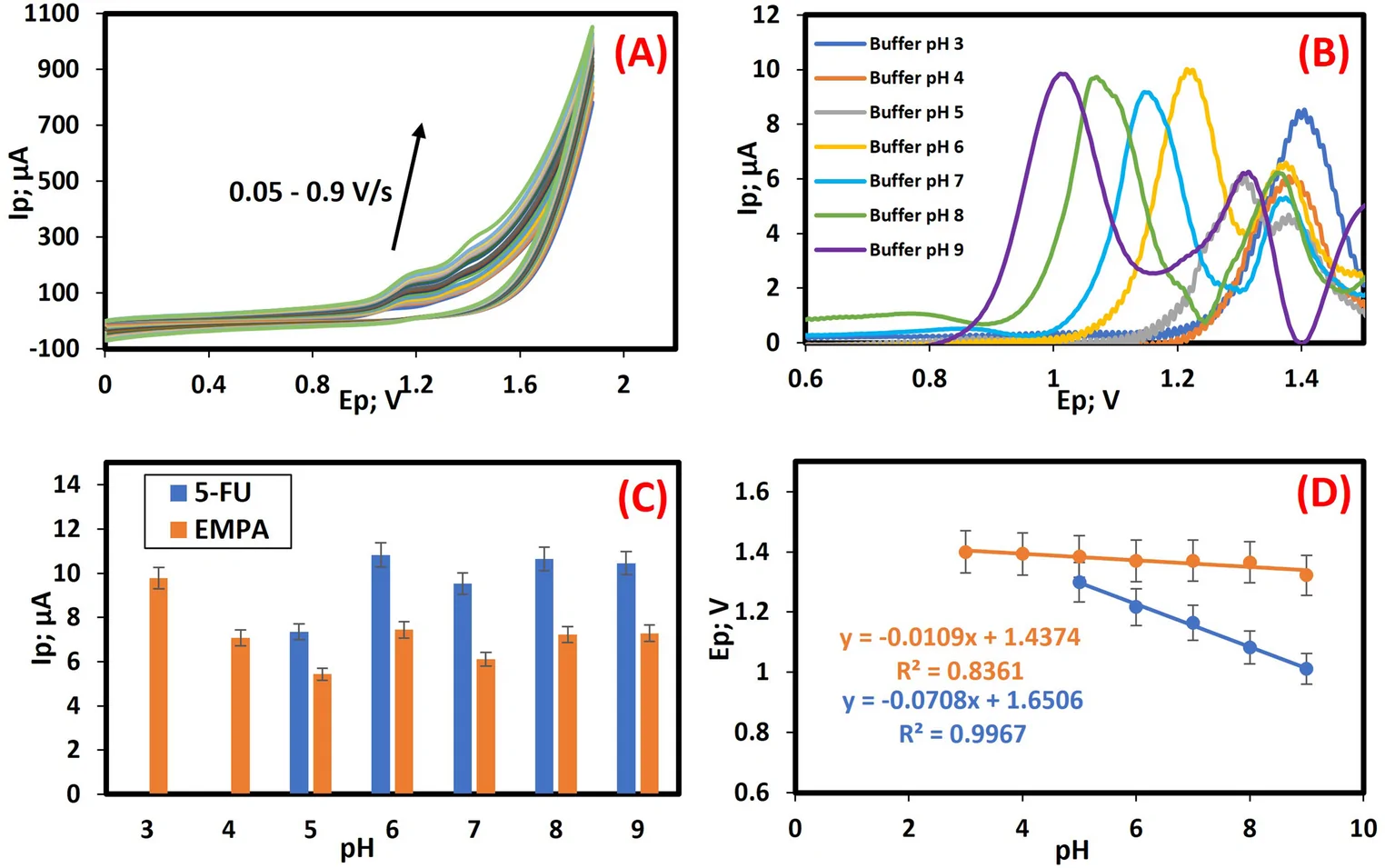
Panel (**A**): The effect of different scan rates (0.05–0.9 V s^-1^) on cyclic voltammograms of 1.05 × 10− 4 M, 5.3 × 10− 5 M of 5-FU and EMPA, respectively, in Britton-Robinson buffer solution (0.04 M, pH 8.0) employing poly-XO@poly-EUC/PGE sensor. (**B** & **C**) The effect of several pH values of 0.04 M of Britton-Robinson buffer solution on the oxidation of 5-FU (15 μM) and EMPA (7.5 μM) employing poly-XO@poly-EUC/PGE sensor. (**D**) Linear curves of potential (Ep) versus pH of the supporting electrolyte

The effect of scan rate on the electro-oxidation behavior of 5-FU and EMPA was investigated in Britton–Robinson buffer (pH 8.0) using the newly developed poly-XO@poly-EUC/PGE sensor. CV measurements were performed over a scan rate range of 0.025–0.9 V s^− 1^. As illustrated in Fig. 3A, the peak current responses of both analytes increased progressively with increasing scan rate, demonstrating a scan rate–dependent electrochemical progression. Furthermore, plotting the peak current (I_p_) against the scan rate yielded well-defined linear relationships for both 5-FU and EMPA (Fig. 3S, A), as follows:

$$For\;5-FU:\;I_p\;(\mu A)\:=\:48.595\:+\:128.42\;\nu\;(V\;s^{-\:1})\;(r^2\:=\:0.9871)$$

$$For\;EMPA:\;I_p\;(\mu A)\:=\:72.454\:+\:218.61\;\nu\;(V\;s^{-\:1})\;(r^2\:=\:0.9903)$$

As shown in Fig. 3A, neither of the two target analytes exhibited cathodic peaks during the reverse scans, confirming the irreversible nature of their electrochemical oxidation. In addition, a positive shift in the anodic peak potentials was noticed with increasing scan rate, further supporting the irreversible oxidation behavior of both 5-FU and EMPA.

Moreover, linear relationships were obtained between the logarithm of the scan rate (log *ν*) and the logarithm of the current intensity (log I_p_) for both analytes, as illustrated in Fig. 3S, B. These relationships can be expressed by these regression equations:   

$$For\;5-FU:\;\log\;I_p\;(\mu A)\:=\:2.2051\:+\:0.4602\;\log\;\nu\;(V\;s^{-\:1})\;(r^2\:=\:0.9918)$$

 

$$For\;EMPA:\;\log\;I_p\;(\mu A)\:=\:2.4243\:+\:0.4935\;\log\;\nu\;(V\;s^{-\:1})\;(r^2\:=\:0.9881)$$

Based on the Randles–Ševčík concept [72], the theoretical slope of the linear relationship between log I_p_ and log *ν* is 0.5. In the present study, the experimentally obtained slopes were 0.4602 for 5-FU and 0.4953 for EMPA. The close agreement between the experimental and theoretical values signifies that the electro-oxidation of both analytes is predominantly controlled by a diffusion-controlled mechanism. These findings are in good agreement with earlier reported electroanalytical studies for the investigated compounds [29, 33, 34, 73].

A linear dependency of I_p_ on v^½^ indicate a diffusion-controlled mechanism, according to the Randles–Ševčík equation assigned for irreversible systems [72]. As revealed in I_p_ vs. v^½^ plots, the generated plots showed good linear correlations (Fig. 3S, C) as follows:

$$For\;5-FU:\;\;I_p\;(\mu A)\;=\;10.981\;+\;147.81\;\nu^\frac12\;{(V\;s^{-1})}^\frac12\;\;(r^2\;=\;0.9939)$$

$$For\;EMPA:\;I_p(\mu A)\;=\;3.6615+261.54\;\nu^\frac12\;{(Vs^{-1})}^{\frac12\;}\;(r^2=0.9800)$$

Moreover, and as described in Fig. 3S, D, linear relationships with good correlation coefficients were obtained for the analytes under investigation by plotting peak potential (E_p_) versus logarithm of scan rate (log *ν*) and obtaining the regression equations as follows:   

$$For\;5-FU:\;E_p\;(V)\:=\:1.2462\:+\:0.1274\;\log\;\nu\;(V s^{-1})\;(r^2\:=\:0.9751)$$

  

$$For\;EMPA:\;E_p\;(V)\:=\:1.4536\:+\:0.1339\;\log\;\nu\;(V s^{-1})\;(r^2\:=\:0.9807)$$

These relationships can be utilized to compute the number of electrons (n) participated in the oxidation of the target analytes based on Laviron equation [74] and according to the following formula:

$$Slope\;of\;E_p\;against\;\log\;\nu\;plot\:=\:2.2303\;RT/nF\alpha$$

In which, the values of slope are 0.1274 and 0.1339 for 5-FU and EMPA, respectively while R, T, F, α constants are universal gas constant (8.314 J mol^− 1^ K^− 1^), the absolute temperature in Kelvin (298 K), Faraday constant (96,480 C mol^− 1^), and the charge transfer coefficient (0.5 for the irreversible reactions). n is the number of involved electrons in the oxidation process. By adjusting the previously estimated slope values in the preceding equation (Laviron equation), the numbers of involved electrons (n) in the oxidation of 5-FU and EMPA using the novel constructed poly-XO@poly-EUC/PGE sensor were 0.899 and 0.855 (~ 1 electron). Therefore, the electro-oxidation of either 5-FU or EMPA across the surface of the manufactured poly-XO@poly-EUC/PGE sensor involves one electron.

### Computational analyses and reaction mechanisms

**Fig. 4:**
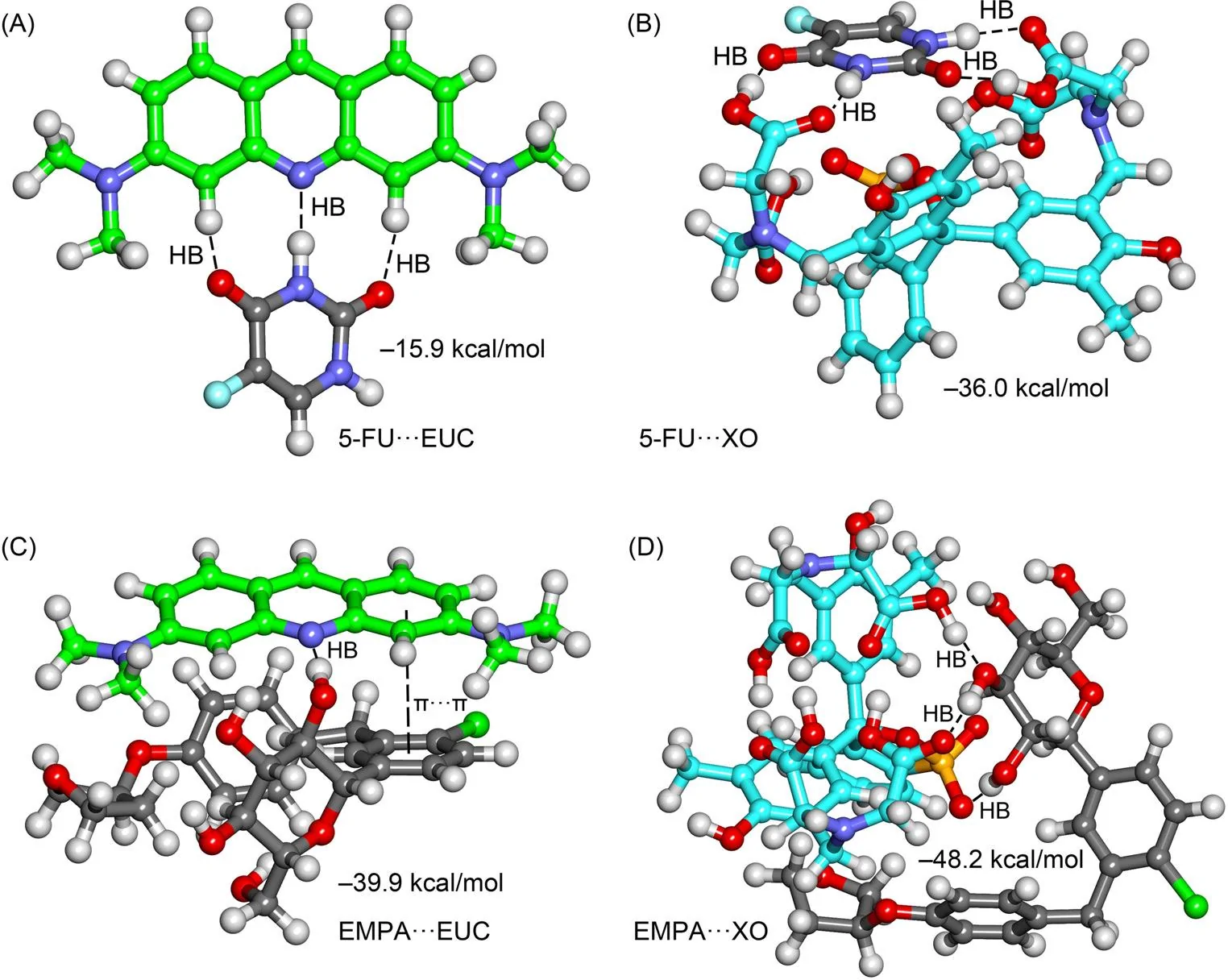
DFT optimized geometries and interaction energies for the 1:1 complexes of the analytes with the monomeric units of the sensing interface: (**A**) 5-FU···EUC, (**B**) 5-FU···XO, (**C**) EMPA···EUC, and (**D**) EMPA···XO. Hydrogen bonding (HB) and π···π stacking interactions are indicated by dashed lines. Theoretical calculations were performed at the BP86-D4/def2-TZVP level of theory. EUC is represented in green and XO in blue

To evaluate the mechanistic interactions governing the sensing behavior of the dual-polymer interface, computational modeling of the 1:1 complexes formed between the analytes and the monomeric units of the modifiers was carried out. The optimized structures and their corresponding interaction energies attained at the BP86-D4/def2-TZVP level of theory are illustrated in Fig. 4. The 5-FU···EUC complex displays an interaction energy of -15.9 kcal/mol, which is established through three hydrogen bonds. Specifically, the central nitrogen atom of the acridine core of EUC dye acts as a hydrogen bond acceptor for the acidic NH group of 5-FU, while the peripheral carbonyl groups of the analyte form complementary hydrogen bonding interactions with the adjacent aromatic CH protons of the EUC ring. In contrast, the 5-FU···XO complex exhibits a significantly stronger interaction energy of -36.0 kcal/mol. This enhanced stability is attributed to the polybasic carboxylic acid and phenolic groups of XO dye, which participate in a network of four strong hydrogen bonds with the imide and carbonyl groups of the 5-FU molecule. A similar trend is observed for the interactions involving EMPA. The EMPA···EUC complex possesses an interaction energy of -39.9 kcal/mol. This association is stabilized by a combination of a hydrogen bond between an aliphatic hydroxyl group of the sugar moiety and the acridine nitrogen atom, along with significant π···π stacking interactions and long range van der Waals interactions between the aromatic ring of the analyte and the extended conjugated system of the EUC core. The strongest interaction among all modeled configurations is found in the EMPA···XO complex, which yields a binding energy of -48.2 kcal/mol. This robust affinity is driven by a complex network of multiple hydrogen bonds between the polyhydroxy sugar domain of the drug and the diverse oxygen-containing functional groups of XO, including its sulfonate and carboxylate centers. The computational findings correlate well with the sequential fabrication and the observed electrochemical behavior of the poly-XO@poly-EUC/PGE sensor. The deposition of XO as the outer layer over the underlying EUC film ensures that the functional groups responsible for the strongest binding affinities are fully exposed to the solution interface. This structural arrangement maximizes the noncovalent entrapment of the target analytes during the preconcentration step, directly facilitating the electrocatalytic signal enhancement observed for the dual-polymer sensor relative to the single-polymer modifications. Furthermore, the calculated interaction energies reveal a distinct thermodynamic preference for EMPA over 5-FU for both polymer models. This higher binding affinity is in excellent agreement with the experimental performance indicators of the SWV method. Thermodynamically favored interactions at the electrode surface typically translate into enhanced operational sensitivity, lower mass-transfer resistance, and more efficient signal transduction. This is directly reflected in the validated analytical parameters, where the proposed sensing platform demonstrates a remarkably lower limit of detection for EMPA (0.02 μM) compared to that obtained for 5-FU (0.33 μM).

**Fig. 5:**
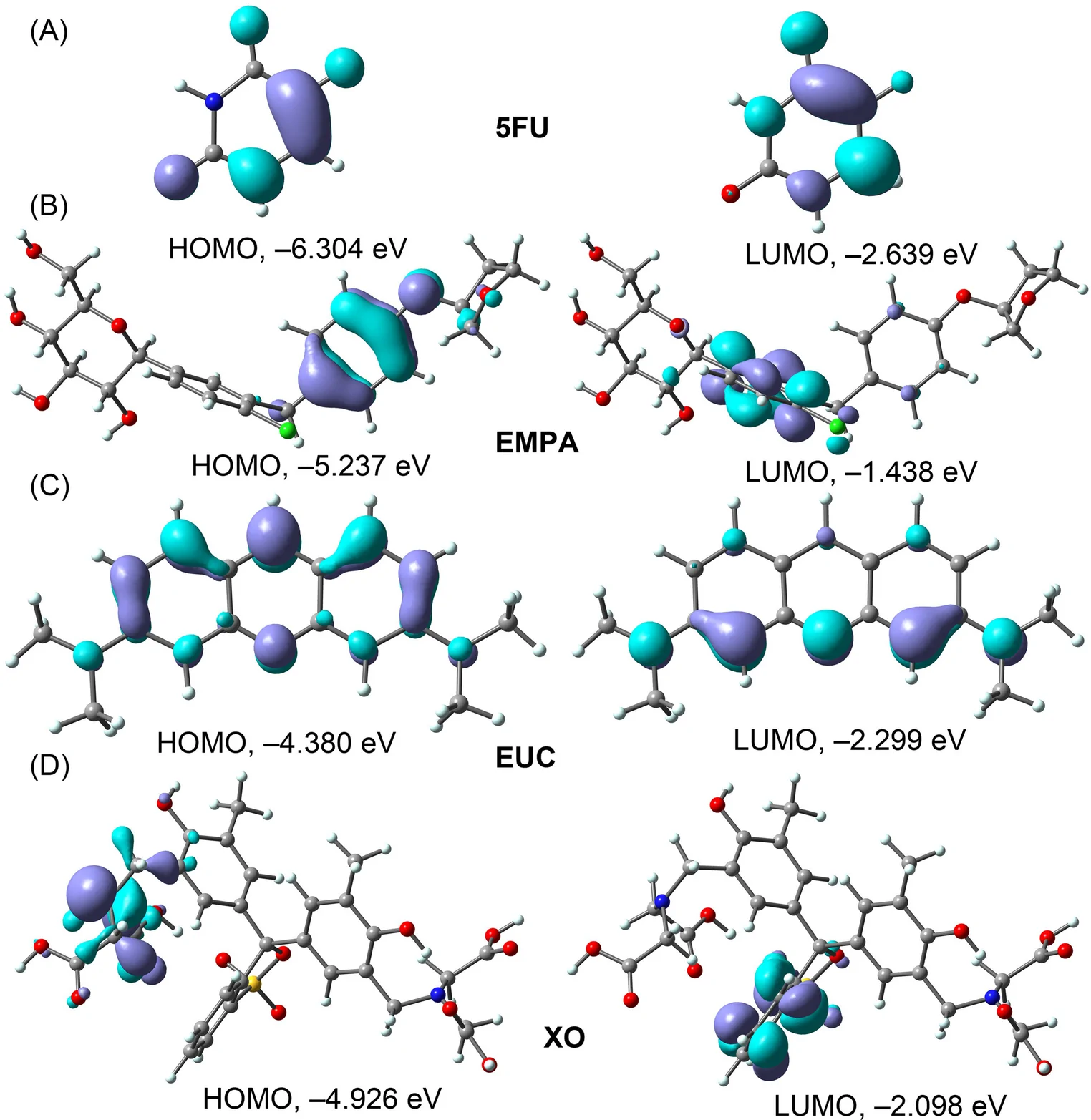
Frontier molecular orbitals, including the highest occupied molecular orbitals (HOMO) and lowest unoccupied molecular orbitals (LUMO), along with their corresponding energy levels in electron volts (eV) for the isolated species calculated at the BP86-D4/def2-TZVP level of theory: (**A**) 5-fluorouracil, (**B**) empagliflozin, (**C**) euchrysine, and (**D**) xylenol orange

To evaluate the electronic properties and gain insight into the electrochemical oxidation mechanisms of the target analytes at the modified interface, the frontier molecular orbitals (FMOs) of all isolated species were analyzed. The highest occupied molecular orbitals (HOMOs) and lowest unoccupied molecular orbital (LUMOs), along with their corresponding electronic energies, are illustrated in Fig. 5. For 5-FU, the highest occupied molecular orbital (HOMO) is located over the pyrimidine ring with contributions from the carbonyl oxygen and fluorine atoms, yielding an orbital energy of -6.304 eV. For EMPA, the HOMO is concentrated on the central chlorobenzyl aromatic moiety with an energy of -5.237 eV, whereas its LUMO is delocalized over the adjacent oxolanyloxy-substituted benzene ring with an energy of − 1.438 eV. In both analytes, the FMOs are predominantly π-type in nature, exhibiting delocalization across the aromatic or heterocyclic frameworks with electronic density extending toward the exocyclic heteroatoms.

Regarding the electrode modifiers, the FMOs of EUC represent a highly conjugated π-system distributed over the tricyclic acridine core, with noticeable delocalization extending into the peripheral exocyclic dimethylamino nitrogen atoms. The HOMO and LUMO energies of EUC are − 4.380 eV and − 2.299 eV, correspondingly. In contrast, the XO monomer displays a localized electronic distribution, where its HOMO is mainly positioned on the lone pair of one of the tertiary amino nitrogen atoms belonging to the iminodiacetic acid domain, with an energy of − 4.926 eV. Its lowest unoccupied molecular orbital, located at − 2.098 eV, is centered over the triphenylmethane ring system and the adjacent sulfonate group.

The calculated HOMO energy of EMPA is higher than that of 5-FU, which theoretically indicates a greater intrinsic electron-donating capability for the isolated EMPA molecule. However, this trend does not align directly with the experimental oxidation potentials, where 5-FU oxidizes at a lower potential than EMPA. This divergence indicates that the electro-oxidation processes on the poly-XO@poly-EUC/PGE sensor are significantly influenced by interfacial kinetic factors, mass-transfer characteristics, and overpotentials associated with the structural reorganization of the larger EMPA framework during the one-electron transfer step, rather than being determined solely by the isolated electronic ground states.

**Fig. 6:**
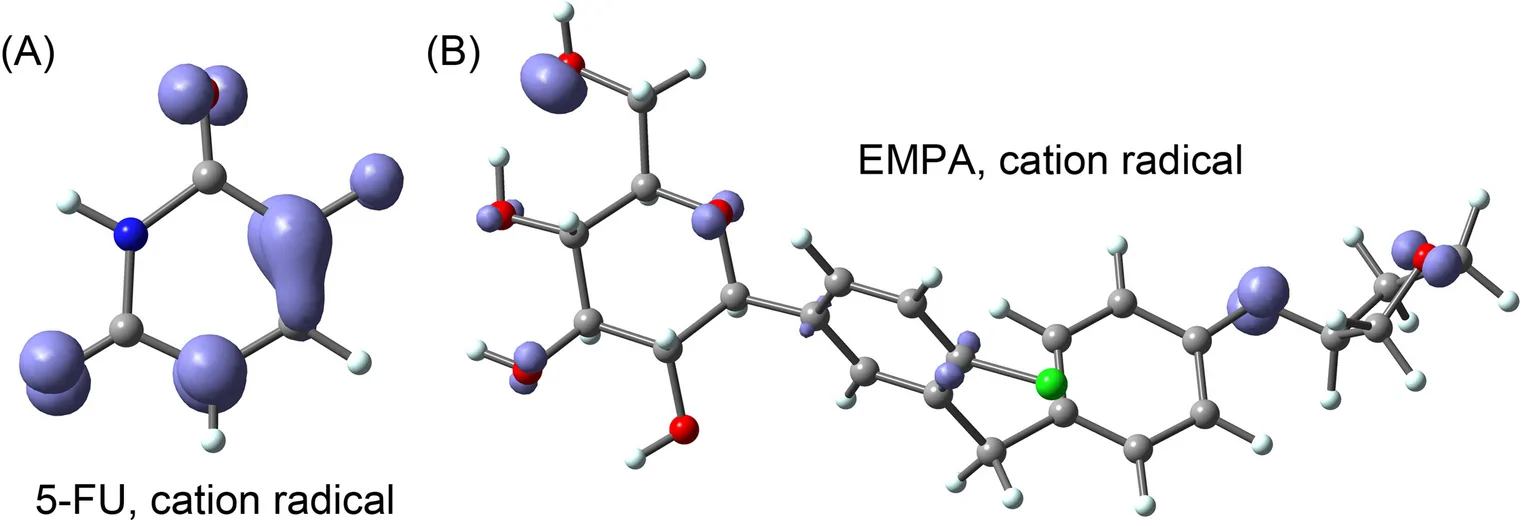
Calculated spin density isosurfaces for the open-shell one- electron oxidation intermediates at the BP86-D4/def2-TZVP level of theory: (**A**) 5-fluorouracil cation radical and (**B**) empagliflozin cation radical. The blue shaded envelopes represent the spatial distribution of the unpaired electron density across the respective molecular frameworks

To further elucidate the radical pathways and validate the atomic sites actively participating in the irreversible electrochemical oxidation, the spin density distributions of the open-shell cation radical intermediates generated after the initial one-electron transfer step were calculated. The resulting spin density isosurfaces for both 5-FU and EMPA are displayed in Fig. 6. For the 5-FU cation radical, the spin density distribution strongly mirrors its HOMO profile, demonstrating that electron removal occurs from a highly delocalized conjugated network. The radical character is distributed across the pyrimidine ring framework and several exocyclic heteroatoms, with the largest spin density concentrated on the ring carbon atom directly adjacent to the fluorine atom. This localization patterns the typical reactivity of the pyrimidine core during anodic oxidation. In contrast, the spin density distribution of the EMPA cation radical exhibits a significant electronic reorganization when compared directly to its ground-state highest occupied molecular orbital. While the highest occupied molecular orbital of the neutral molecule is predominantly centered on the chlorobenzyl ring system, the spin density of the resulting radical cation shifts dramatically toward the oxygen atom centers. In particular, a major portion of the spin density is highly localized on the oxygen atom of the exocyclic hydroxymethyl group attached to the oxane sugar ring.

**Scheme 1:**
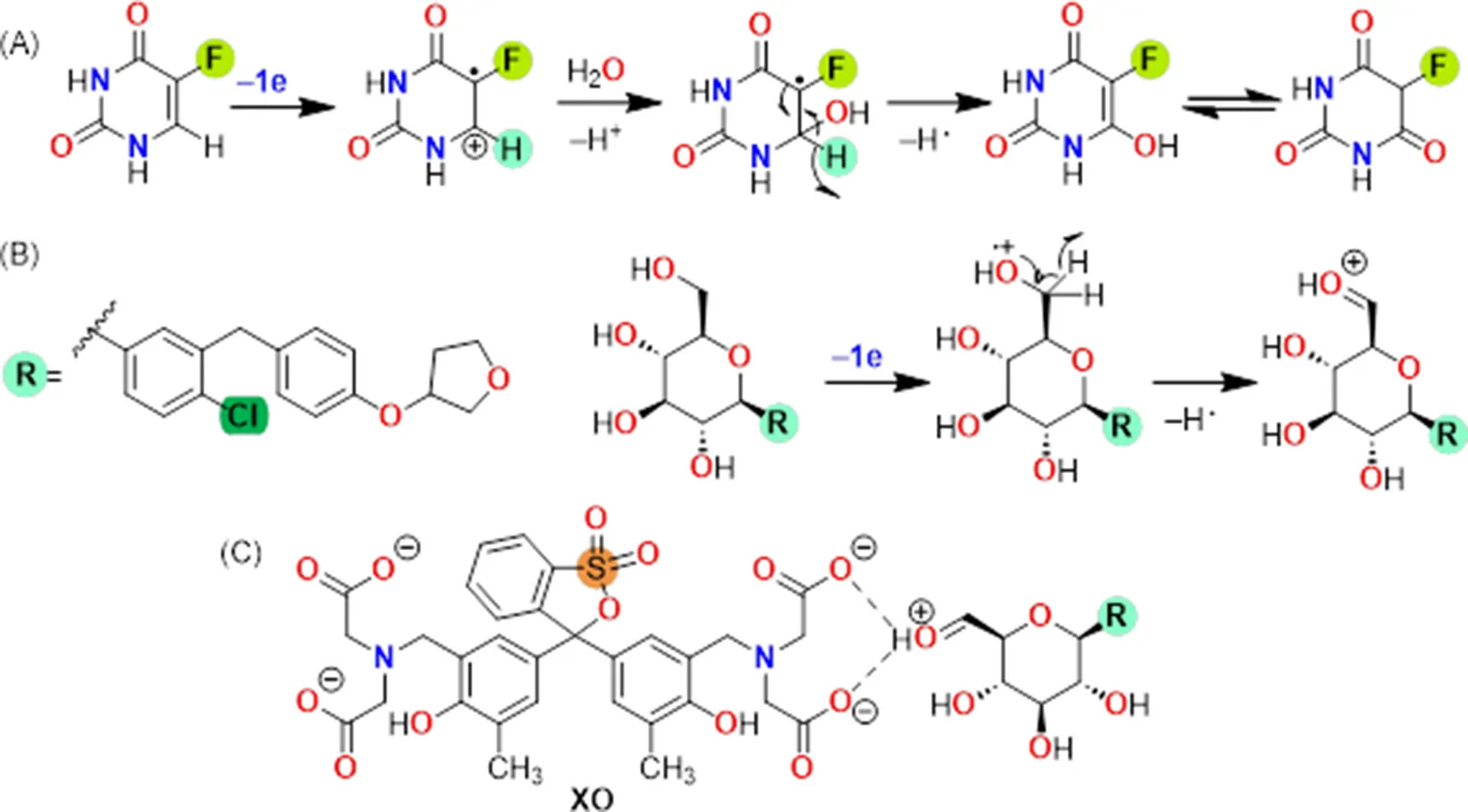
Proposed electrochemical oxidation mechanisms over the modified dual-polymer sensing platform: (**A**) 5-FU pathway yielding the tautomeric trione derivative via a water-mediated nucleophilic step, and (**B**) EMPA pathway demonstrating the conversion of the primary exocyclic hydroxymethyl group into an aldehyde derivative through a sequential one-electron radical intermediate cascade. (**C**) Proposed interaction of the poly-XO with the oxocarbenium oxidized product

To provide a comprehensive mechanistic rationale for the anodic processes, a detailed electrochemical oxidation pathway for both analytes is proposed as illustrated in Scheme 1. The reaction mechanisms are fully supported by the experimental verification of a one-electron transfer process, the calculated computational spin density distributions of the open-shell intermediates, and established electroanalytical literature for related structures [33]. For 5-FU, the initial one-electron oxidation step removes an electron from the delocalized framework to produce a highly reactive and electrophilic radical cation intermediate. This species undergoes nucleophilic attack by a water molecule from the aqueous solvent matrix with the simultaneous loss of a proton, successfully introducing a hydroxyl group at the carbon 6 position adjacent to the fluorine atom. Subsequent abstraction of a hydrogen radical breaks the carbon-hydrogen bond at this center, yielding 5-fluoro-6-hydroxyuracil, which exists in a dynamic keto-enol tautomeric equilibrium with its corresponding 5-fluoropyrimidinetrione form. For EMPA, the oxidation mechanism targets the primary aliphatic alcohol domain, as independently confirmed by the dramatic localization of spin density on the exocyclic hydroxymethyl group of the sugar ring. The initial one-electron abstraction yields an oxygen-centered cation radical intermediate localized on this specific hydroxyl site. Following the formation of this highly unstable intermediate, the extraction of a hydrogen radical from the adjacent carbon atom takes place, generating a protonated oxocarbenium species. The positive charge of the protonated product is strongly stabilized by the negative charges of the poly-XO dye, as represented in Scheme 1C. This sequential one-electron and hydrogen radical elimination process provides a consistent thermodynamic and structural explanation for the irreversible anodic response observed experimentally for the EMPA molecule at the poly-XO@poly-EUC/PGE interface.

### Electroanalysis of 5-FU and EMPA by the developed SWV methodology

#### Method optimization

The instrumental and experimental factors of the SWV technique for the electrochemical oxidation of a 5-FU and EMPA mixture in Britton–Robinson buffer (0.04 M) were optimized using the newly constructed poly-XO@poly-EUC/PGE sensor.

##### Effect of pH

The electro-oxidation behavior of the target analytes is powerfully influenced by the type and pH of the supporting electrolyte. Hence, different buffer systems and pH values were systematically investigated with respect to the peak current responses of both 5-FU and EMPA. Regarding buffer selection, Britton–Robinson, phosphate, and borate buffers were evaluated, and Britton–Robinson buffer provided the highest anodic peak currents for both analytes, as shown in Fig. 4S. Accordingly, Britton–Robinson buffer (0.04 M) was selected for further studies.

The influence of pH was then inspected over the range of 3.0–9.0 to determine the optimal analytical conditions. As illustrated in Fig. 3B and C, the square-wave voltammograms revealed two well-defined peaks for 5-FU and EMPA when the pH was increased from 5.0 to 9.0. However, only a single peak corresponding to EMPA was observed in the pH range of 3.0–4.0. With rising pH from 3.0 to 6.0, the peak current responses of both analytes increased, followed by a decrease at pH 7.0, and then an increase again at pH 8.0. An additional increase in pH beyond 8.0 resulted in a decline in the peak currents of both 5-FU and EMPA.

Although pH 6.0 provided a smaller peak-to-peak separation (ΔE = 0.156 V), pH 8.0 was chosen as the best condition due to the significantly improved peak separation and resolution (ΔE = 0.280 V), which is more suitable for simultaneous determination. Therefore, Britton–Robinson buffer (0.04 M, pH 8.0) was elected as the optimum supporting electrolyte. Additionally, the relationship between anodic peak potential and pH of the Britton–Robinson buffer was investigated, revealing a gradual shift of the peak potentials toward less positive values with increasing pH, as illustrated in Fig. 3D and described by the following equations:   

$$For\;5-FU:\;E_p\;=\;-0.0708\;pH\:+\:1.6506\;(r^2\:=\:0.9967)$$

  

$$For\;EMPA:\;E_p\;=\;-0.0109\;pH\:+\:1.4374\;(r^2\:=\:0.8361)$$

With respect to the slopes derived from the linear relationship between oxidation peak potential and pH, the slope attained for 5-FU (− 0.0708 V/pH) was close to the theoretic value of − 0.059 V/pH, demonstrating that the electro-oxidation process of 5-FU comprises an equal number of electrons and protons (1e^−^/1H^+^ mechanism). In contrast, the slope observed for EMPA (-0.0109 V/pH) deviated significantly from the theoretical value, suggesting that protons are not involved in its electro-oxidation process.

##### Instrumental parameters

The instrumental parameters of the developed SWV method, including step potential, pulse amplitude, and frequency, were systematically optimized as shown in Fig. 5S. The effect of step potential was studied over the range of 1–15 mV. The peak current responses of both 5-FU and EMPA increased with increasing step size up to 7 mV, which provided the maximum anodic peak currents. However, further increases in step potential (≥ 10 mV) led to a noticeable decrease in signal intensity. Accordingly, a step potential of 7 mV was nominated as the optimum value (Fig. 5S, A and B).

The pulse amplitude was also inspected within the range of 1–10 mV. The peak currents of 5-FU and EMPA increased progressively with increasing pulse amplitude up to 10 mV. Beyond this value, the voltammetric peaks became distorted, indicating a loss of signal quality. Therefore, a pulse amplitude of 10 mV was chosen for subsequent measurements (Fig. 6S, C and D).

Finally, the influence of frequency was tested over the range of 5–17 Hz. The current responses increased with frequency up to 12 Hz, which yielded the highest peak intensities for both analytes. At higher frequencies (≥ 15 Hz), the peak currents decreased, likely due to insufficient interaction time at the electrode surface. Consequently, 12 Hz was chosen as the best frequency for further experimentations (Fig. 6S, E and F).

#### Analytical method validation

The developed SWV methodology for the simultaneous determination of 5-FU and EMPA in their bulk forms and plasma samples was validated to evaluate linearity, limits of detection and quantitation, accuracy, precision, and selectivity in agreement with ICH [59] and EMA [60] guidelines.

##### Linearity and sensitivity limits

**Fig. 7:**
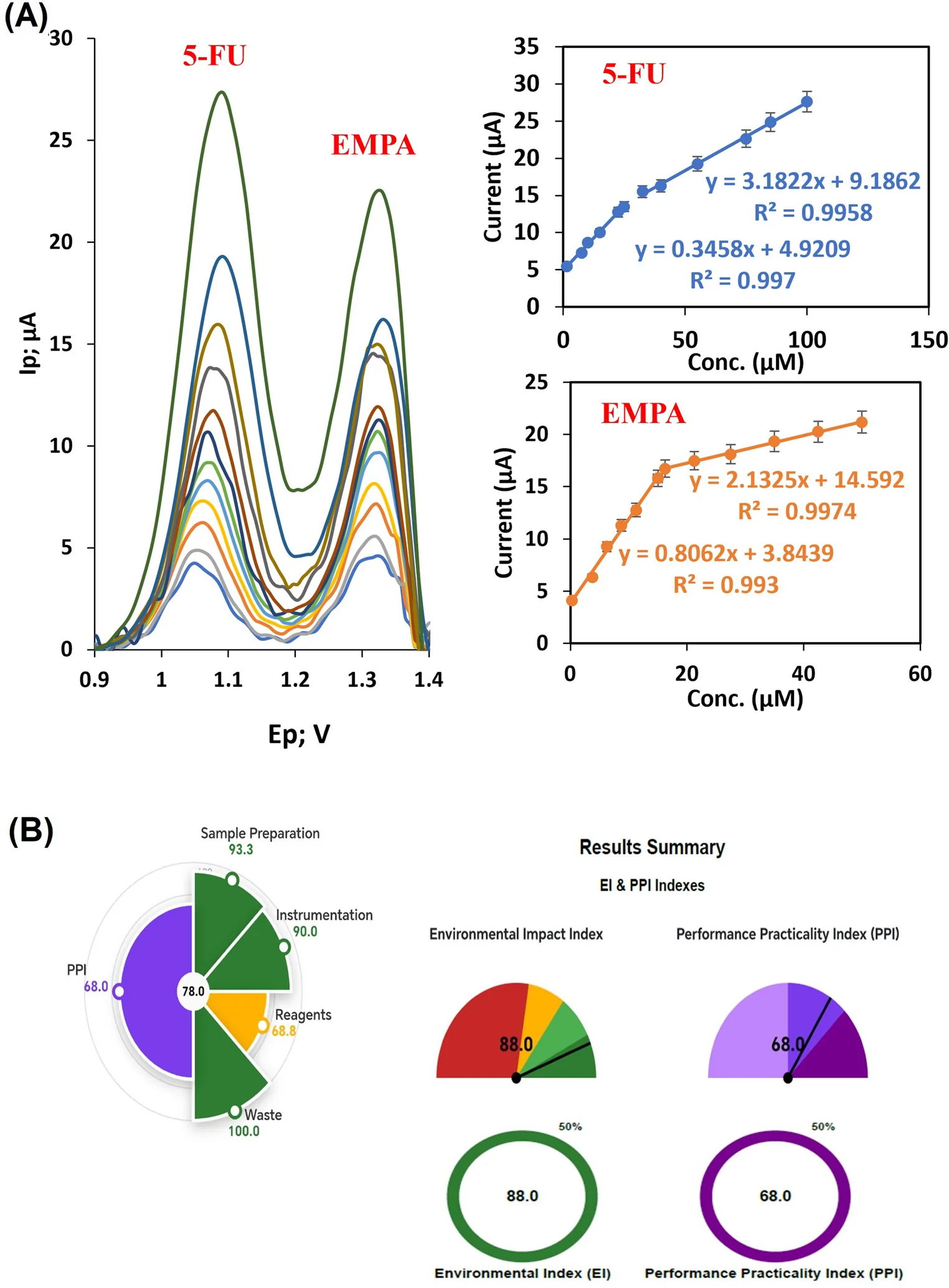
Panel (**A**): SW curves of different concentrations of 5-FU and EMPA in Britton-Robinson buffer (0.04 M, pH 8.0) using poly-XO@poly-EUC/PGE sensor, (Inset) the corresponding calibration lines at two linear ranges for 5-FU (1.50–25.0 µM and 32.50–100.0 µM) and EMPA (0.25–15.0 µM and 16.25–50.0 µM) at scan rate 0.1 V s^− 1^. Panel (**B**): The EPPI images for the evaluation of sustainability parameters of the developed SWV method for simultaneous determination of 5-FU/EMPA mixture

Using the newly developed SWV method, the quantitative relationship between the anodic peak currents of 5-FU and EMPA and their corresponding concentrations was assessed under optimized experimental conditions. The SWV responses recorded at the poly-XO@poly-EUC/PGE sensor for different concentrations of 5-FU and EMPA are presented in Fig. 7A. A gradual increase in peak current was observed with increasing analyte concentration, resulting in well-defined linear calibration plots (Fig. 7A, inset).

As summarized in Table 1, two distinct linear dynamic ranges were obtained for 5-FU (1.50–25.0 µM and 32.5–100.0 µM), while EMPA also exhibited two linear ranges (0.25–15.0 µM and 16.25–50.0 µM). The excellent linearity of the developed SWV method is further confirmed by the good correlation and determination coefficients obtained for both analytes.

**Table 1:** Statistical results for the determination of 5-FU and EMPA mixture by the proposed SWV method using the poly-XO@poly-EUC/PGE probe

Statistical parameter	5-FU			EMPA	
	Range 1	Range 2		Range 1	Range 2
Linearity range (µM)	1.50–25.0	32.50–100.0	0.25–15.0		16.25–50.0
Correlation coefficient (r) ± SD^a^	0.9985 ± 0.0014	0.9992 ± 0.0012	0.9965 ± 0.0014		0.9987 ± 0.0012
Determination coefficient (r^2^) ± SD^a^	0.9970 ± 0.0028	0.9958 ± 0.0024	0.9930 ± 0.0028		0.9974 ± 0.0024
Intercept (a) ± SD^a^	4.92 ± 0.035	9.19 ± 0.295	3.84 ± 0.005		14.38 ± 0.388
Slope (b) ± SD^a^	0.35 ± 0.006	3.18 ± 0.006	0.81 ± 0.007		2.13 ± 0.124
LOD^b^ (µM)	0.33 (42.9 ng/mL)		0.02 (9.0 ng/mL)		
LOQ^b^ (µM)	1.01 (131.3 ng/mL)		0.062 (27.9 ng/mL)		

^a^ Average of five replicates

^b^ Limits of detection (LOD) and quantitation (LOQ)

The limits of detection (LOD) and quantitation (LOQ) were calculated via the typical equations: LOD = 3.3σ/slope and LOQ = 10σ/slope, whereas σ represents the standard deviation of the calibration curve intercept. For the calculation of LODs and LOQs, 0.35 and 0.035 are the estimated values of slope and σ, respectively, used for computing the limits of 5-FU. While the estimated values of slope and σ used for calculation of LOD and LOQ of EMPA are 0.81, 0.005, respectively.

Based on these computations, the LODs and LOQs were found to be 0.33 µM (42.9 ng/mL) and 1.01 µM (131.3 ng/mL) for 5-FU, and 0.02 µM (9.0 ng/mL) and 0.062 µM (27.9 ng/mL) for EMPA, respectively. These findings demonstrate the high sensitivity of the established method for the electrochemical analysis of the investigated medications.

The observation of two distinct linear calibration ranges does not necessarily indicate an experimental artifact; rather, it may reflect a concentration-dependent change in the dominant electrochemical or mass-transfer process. This behavior can plausibly arise from changes in mass transport and/or analyte–electrode interactions. Two possible explanations are considered:

1. Transition in the mass-transfer process:

At lower analyte concentrations, the electrochemical response may predominantly follow diffusion-controlled behavior, with the peak current increasing proportionally with analyte concentration according to the Randles–Ševčík relationship (I_p_ = 0.4463nFAC (nFD/RT)^½^) where the peak current is proportional to the analyte concentration, giving rise to the first linear calibration region [75]. As the analyte concentration increases, the rate of electrochemical consumption at the electrode surface may increase relative to the rate of replenishment from the bulk solution. Consequently, changes in the concentration gradient and diffusion-layer characteristics may alter the mass-transfer regime. Under these conditions, the current response may deviate from the ideal Randles–Ševčík behavior, resulting in a different calibration slope [76, 77]. The distinct slopes observed in the two concentration regions therefore suggest a concentration-dependent change in the overall mass-transfer/electrode-response characteristics.

2. Concentration-dependent adsorption of the analyte:

A second possible contribution is the concentration-dependent interaction of the analyte with the modified electrode surface. At relatively low concentrations, adsorption or surface interactions may contribute appreciably to the electrochemical response, effectively increasing the local analyte concentration at the interface and thereby enhancing the analytical sensitivity following a Langmuir isotherm [78]. With increasing analyte concentration, progressive occupation of available surface sites may reduce the contribution of further surface accumulation. The response may then become increasingly governed by transport of the analyte from the bulk solution to the electrode surface, leading to a change in the calibration slope. In the present study, the sharp and well-defined oxidation peaks observed in the higher-concentration region, together with the altered calibration slope, are consistent with a greater contribution from diffusion-controlled mass transport in this concentration range.

Nevertheless, because the present experiments do not directly distinguish between these possible contributions, the observed two-range behavior is conservatively attributed to a concentration-dependent change in the interfacial mass-transfer and/or analyte–electrode interaction, rather than assigning it exclusively to a single mechanism.

##### Accuracy and precision

According to ICH recommendations, accuracy and precision are essential parameters for method validation. The accuracy and precision of the developed SWV methodology for the determination of 5-FU and EMPA were evaluated at three concentration levels (low, medium, and high), including both intra-day and inter-day studies. The outcomes are summarized in Table 2.

**Table 2:** Accuracy and precision of the developed SWV method for determination of the studied 5-FU/EMPA mixture employing poly-XO@poly-EUC/PGE sensor

Drug	Concentration (µM)	Accuracy		Intra-day precision		Inter-day precision	
		% Recovery ± SD^a^	RSD%	% Recovery ± SD^a^	RSD%	% Recovery ± SD^a^	RSD%
5-FU	1.5	97.85 ± 0.55	0.56	98.16 ± 0.99	1.01	103.59 ± 0.84	0.81
	40.0	100.26 ± 1.08	1.08	99.74 ± 0.53	0.53	102.45 ± 0.66	0.64
	100.0	101.78 ± 1.12	1.10	98.23 ± 0.75	0.76	101.42 ± 0.43	0.42
EMPA	0.25	100.02 ± 0.56	0.56	102.78 ± 0.45	0.44	102.10 ± 0.53	0.52
	20.0	98.45 ± 0.85	0.86	100.56 ± 0.75	0.75	99.11 ± 0.93	0.94
	50.0	96.21 ± 1.14	1.18	100.89 ± 1.52	1.51	99.35 ± 1.68	1.69

^a^ Average of six replicates and the results were compared with those of the calibration lines

Regarding accuracy, acceptable recovery values were obtained from six replicate measurements at each concentration level, ranging from 96.21% to 101.78%, with relative standard deviation (RSD%) values below 1.18%, indicating good agreement between the measured and nominal concentrations. In addition, intra-day and inter-day precision were measured at the same concentration levels over one day and three successive days, individually. The recovery percentages ranged from 98.16% to 103.59%, with satisfactory RSD% values between 0.42% and 1.69%.

Overall, these results demonstrate that the developed SWV method is both accurate and precise for the concurrent determination of 5-FU and EMPA.

##### Robustness

Robustness, defined as the evaluation of the method’s reliability under small deliberate variations in analytical parameters, is an important validation criterion. Accordingly, the robustness of the proposed SWV methodology was assessed by introducing slight variations in experimental conditions, including the pH of the Britton–Robinson buffer, as well as instrumental parameters such as step potential, pulse amplitude, and frequency.

As shown in Table 1S, minor variations in these parameters had negligible effects on the anodic peak currents of both 5-FU and EMPA, with recovery values ranging from 97.22% to 103.80% and RSD% values below 1.31%. The results confirm the robustness and reliability of the established SWV method under small operational changes.

##### Interference-based selectivity

Under optimized conditions, the selectivity of the proposed SWV procedure for the concurrent determination of 5-FU and EMPA was evaluated in the existence of various potential interfering substances. These included common excipients such as starch, NaCl, and MgCl_2_, as well as co-administered drugs used either to manage 5-FU-associated side effects (e.g., ondansetron and dexamethasone) or drugs that may be administered concurrently (e.g., sitagliptin, pioglitazone, and metformin).

Typically, the interfering species were added at concentrations 10-fold higher than that of 5-FU and 20-fold higher than that of EMPA. The effect of these interferents was assessed by calculating the recovery percentages of each analyte in their presence. As presented in Table 2S, the recovery percentages ranged from 96.38% to 102.37%, with RSD% values below 2.05%. These results confirm the excellent interference-based selectivity of the developed SWV methodology for the concurrent determination of 5-FU and EMPA.

##### Reproducibility and stability of poly-XO@poly-EUC/PGE

Six independently prepared poly-XO@poly-EUC/PGE sensors, constructed under identical circumstances, were used to assess the repeatability of the proposed probe prior to its application for 5-FU and EMPA determination. As shown in Fig. 6S, A, the calculated recovery values for the parallel-modified electrodes ranged from 97.62% to 101.87%, with RSDs between 0.65% and 1.51%, confirming the good reproducibility of the fabricated sensor.

Additionally, the stability of the modified electrode was assessed over a period of 15 days for the determination of both analytes. During this storage period, the oxidation peak currents of 5-FU and EMPA remained nearly constant, with recovery values ranging from 96.31% to 102.84%, as illustrated in Fig. 4S, B. These results demonstrate the good long-term stability of the developed poly-XO@poly-EUC/PGE sensor.

### Application of the SWV approach for assessment of 5-FU and EMPA in plasma

The acceptable sensitivity and selectivity of the developed sensor enabled successful analysis of 5-FU and EMPA in human plasma using the proposed SWV method. The reliability of the method for quantifying both analytes in plasma samples was confirmed by good recovery values ranging from 98.75% to 101.33%, with RSD% values below 1.27%, ensuing optimization of the extraction procedure and employment of the analytical procedure (Sect. Simultaneous determination of 5-FU and EMPA in human plasma) (Table 3).

**Table 3:** Assay of 5-FU and EMPA in spiked human plasma by the developed SWV method

Analyte	Claimed concentration (µM)	Found concentration (µM)	% Recovery* ± SD	RSD%
5-FU	1.5	1.52	101.33 ± 0.75	0.74
	40.0	39.50	98.75 ± 1.25	1.27
	100.0	100.01	100.01 ± 1.05	1.05
EMPA	0.25	0.248	99.20 ± 0.45	0.45
	40.0	40.10	100.25 ± 0.65	0.65
	50.0	49.99	99.98 ± 0.68	0.67

^*^ Average of three measurements

Furthermore, statistical comparison with previously reported methods [15, 34] was performed at the 95% confidence level using Student’s t-test and F-test. The determined t-values (1.333 for 5-FU and 2.374 for EMPA) and F-values (0.348 and 0.605, respectively) were lower than the corresponding theoretic values, representing the absence of any significant difference between the proposed SWV method and the reference methods (Table 4).

**Table 4:** Statistical comparison between the developed SWV method and reported methods

Analyte	Recovery (% ± SD) ^a^		Calculated values ^b^		
	SWV method	Reported method	t-value	F-value	*P*-value
5-FU	99.89 ± 0.85	98.98 ± 1.44 [15]	1.333	0.348	0.212
EMPA	101.45 ± 0.56	100.20 ± 0.72 [34]	2.374	0.605	0.077

^a^ Mean of 6 determinations for 5-FU and 3 determinations of EMPA

^b^ Theoretic t- and F- values at 95% confidence limit are 2.228 and 5.053, individually for 6 determinations whereas for 3 determinations, t- and F- values are 2.776, 19.00, respectively

## Sustainability evaluation

The developed SWV method based on the poly(XO)@poly(EUC)/PGE showed exceptional sustainability and analytical applicability in the Environmental, Practicality, and Performance Index (EPPI) evaluation. With a high overall EPPI score of 78.0, the approach is highly recommended for routine analysis, see (Fig. 7B**)**.

The suggested sensor is categorized as the best green analytical technique with an Environmental Index (EI) value of 95.0. The maximum scores in sample preparation, reagents, and waste management (100.0 each) are indicative of the straightforward electrode production process, which requires few sample preparation steps and the use of reasonably safe reagents. Furthermore, compared to traditional chromatographic techniques, the established SWV approach inherently lowers solvent usage and waste production.

From a performance standpoint, the sensor’s good applicability for simultaneous determination of 5-FU and EMPA is confirmed by its Performance Practicality Index (PPI) score of 68.0. The requirement for electrochemical workstations may be the reason for the minor decline in the instrumentation score (80.0); however, such equipment is still reasonably priced, readily available, and appropriate for regular laboratory use. Overall, the EPPI evaluation confirms that the suggested electrochemical sensor is both environmentally sustainable and analytically effective, highlighting its potential as a useful and environmentally friendly substitute for the simultaneous determination of the analytes under study.

## Comparative evaluation of the established SWV methods and previous reports

Some of the previously reported approaches for the determination of either 5-FU or EMPA are summarized in Table 3S. Chromatographic [20, 22, 24, 26, 27, 79–81] and spectroscopic [15, 17, 19, 82–84] techniques, compared with the proposed SWV method, suffer from several limitations, with lengthy analysis time, high instrumental and operational costs, extensive consumption of organic solvents with associated environmental worries, and the need for additional derivatization or extraction steps. Although these methods offer advantages such as extraordinary precision, outstanding reproducibility, and strong capability for separating complex media, their drawbacks may limit their applicability in routine and sustainable analysis, leading to increased time and cost of analysis.

In contrast, the developed SWV method overcomes these limitations by requiring only small sample volumes, eliminating the need for derivatization or complicated extraction procedures, and providing high sensitivity and reproducibility, as summarized in Table 3S. Notably, the proposed method exhibits excellent sensitivity, with LOD values of 0.33 µM and 0.02 µM for 5-FU and EMPA, individually.

Furthermore, when compared with other reported voltammetric techniques [28, 30, 33, 35, 36, 73] the developed SWV approach demonstrates clear advantages, including superior sensitivity, simple sensor construction, low cost, rapid analysis time, and reduced usage of harmful chemicals. The reported SWV and dispersive pulse voltammetric (DPV) methods have employed various probes such as pre-treated glassy carbon electrode, ZnO modified carbon paste electrode, and porphyran-capped silver nanoparticles-modified electrode for determination either 5-FU or EMPA in pharmaceutical dosage forms and biological samples. Their sensitivity limits (LODs range: 1.71–10.7 µM) were compared to that of the developed SWV method (0.33, 0.02 µM for 5-FU and EMPA, respectively), indicating higher sensitivity of the proposed methodology, refer to Table 3S.

Overall, the newly developed poly-XO@poly-EUC/PGE sensor represents a promising analytical platform for the simultaneous determination of 5-FU and EMPA, outperforming conventional spectroscopic, chromatographic, and several electrochemical methods.

## Limitations and future perspectives

Although the developed poly-XO@poly-EUC/PGE sensor demonstrated excellent sensitivity, selectivity, sustainability, and applicability for the simultaneous determination of 5-FU and EMPA during their potential concomitant use/investigational combination, several aspects warrant further investigation. The method was validated using spiked plasma samples, and its performance should be further confirmed in authentic clinical samples obtained from either experimental animals under pre-clinical studies or from patients receiving both drugs for concomitant use. Additional pharmacokinetic and clinical studies involving authentic patient samples would be required before the proposed method could be considered for clinical therapeutic monitoring of this drug mixture. On the other hand, although satisfactory interference-based selectivity was achieved, the effects of a broader range of endogenous metabolites, degradation products, and co-administered drugs remain to be systematically evaluated. The long-term operational stability and inter-batch reproducibility of independently fabricated sensors also require assessment under routine laboratory conditions. Furthermore, while DFT calculations provided valuable mechanistic insights into the analyte–sensor interactions and oxidation pathways, complementary experimental techniques such as spectroelectrochemistry or LC–MS-based product identification would provide additional validation of the proposed mechanism. Another limitation of the computational model of the DFT analyses is that the investigations were performed using isolated molecular models of EUC and XO to investigate qualitative analyte–modifier interactions, rather than to reproduce the complete polymerized sensing interface. This model does not explicitly account for the polymeric network. Future computations should be interpreted as a complete polymerized platform not as direct atomistic representation.

Future studies should therefore focus on translating the proposed sensing platform into portable and disposable point-of-care devices for therapeutic drug monitoring. Incorporating advanced nanostructured conductive materials and multiplexed sensing strategies may further improve sensitivity and enable the simultaneous determination of 5-FU-EMPA, their metabolites, and related co-administered drugs.

The integration of machine learning-assisted signal processing could also enhance analytical performance in complex biological matrices. Moreover, further optimization to reduce reagent consumption and evaluation in large-scale routine applications would strengthen the sustainability profile of the method. Finally, the supramolecular sensing strategy developed in this work may provide a versatile platform for designing selective electrochemical sensors for other anticancer drugs and combination therapies.

## Conclusion

A novel poly-XO@poly-EUC/PGE sensor was effectively established and functioned for the sensitive and selective simultaneous electroanalysis of 5-FU, a chemotherapeutic agent associated with cardiotoxicity and nephrotoxicity, and EMPA as a protective agent. The designated sensor was systematically characterized via SEM, CV, and EIS measurements, confirming its enhanced surface morphology, enlarged electroactive surface area, improved charge-transfer kinetics, and superior electrochemical performance toward the oxidation of both analytes. The anticipated SWV methodology was systematically optimized and validated in accordance with ICH and EMA guidelines. Validation results demonstrated excellent linearity, sensitivity, precision, accuracy, selectivity, and robustness. The method exhibited very low detection and quantification limits, enabling trace-level analysis of both drugs. Accuracy and precision studies yielded satisfactory recoveries ranging from 97.28% to 103.59%, with RSD% values not exceeding 1.51%, confirming the high reliability of the method. The applicability of the proposed sensing interface was successfully demonstrated in human plasma samples after a simple extraction procedure, with recovery values indicating excellent analytical performance under biological matrix conditions. The Environmental, Practicality, and Performance Index (EPPI) score was 78.0, indicating the exceptional sustainability and analytical applicability of the developed method for routine analysis.

Comprehensive DFT computations successfully rationalized the supramolecular performance of the sensing interface. Global minimum geometries located via CREST demonstrated that the outer XO polymer layer establishes strong noncovalent hydrogen-bonding pathways with both drugs, showing a stronger thermodynamic binding affinity for EMPA than for 5-FU, matching the lower experimental LOD found for the gliflozin molecule. Furthermore, cation radical spin density profiles confirmed that the irreversible anodic process is a one-electron transfer. Spin densities identified the carbon center next to the fluorine atom in 5-FU and the primary hydroxymethyl domain of the sugar ring in EMPA as the active reactive centers, confirming a clear pathway where the latter transforms into an aldehyde derivative.

Furthermore, statistical comparison with reported methods confirmed the accuracy and equivalence of the proposed approach. Compared with previously reported spectroscopic, chromatographic, and electrochemical techniques, the developed method offers clear advantages, including simplicity, low cost, rapid analysis, minimal sample and reagent consumption, and high sensitivity. To our best knowledge, this is the first electrochemical approach for the concurrent determination of 5-FU and EMPA. Overall, the developed poly-XO@poly-EUC/PGE sensor represents a promising analytical platform for routine, sensitive, reliable, and sustainable analysis of 5-FU and EMPA in diverse matrices. 

## Supplementary Information

Below is the link to the electronic supplementary material.

Supplementary Material (DOCX 1.45 MB)

## Data Availability

Data will be made available on a reasonable request.
