# Supplementary material for: A dual-electropolymerized poly(Xylenol orange)@poly(Euchrysine) sensing interface for sustainable simultaneous electroanalysis of 5-fluorouracil and empagliflozin: density functional theory mechanistic elucidation
Source: Mikrochim Acta. 2026 Oct 2;193(11):711. doi: 10.1007/s00604-026-08402-y (PMC13633346; doi:10.1007/s00604-026-08402-y)
Supplement: Supplementary file 1 [file 604_2026_8402_MOESM1_ESM.docx]

***Supplementary Materials***

**A dual-electropolymerized poly(Xylenol orange)@poly(Euchrysine) sensing interface for sustainable simultaneous electroanalysis of 5-fluorouracil and empagliflozin: DFT mechanistic elucidation**

Rania S. Ibrahim^1^, Hanaa M. Abd El-Wadood^1^, Marwa R. El-Zahry^1,2^, Antonio Frontera^3^,

Noha M. Hosny^1*^

**^1^** Pharmaceutical Analytical Chemistry Department, Faculty of Pharmacy, Assiut University, Assiut 71526, Egypt

^2^ Pharmaceutical Chemistry Department, Faculty of Pharmacy, Badr University in Assiut, Assiut 2014101, Egypt

^3^ Departament de Química, Universitat de les Illes Balears, Crta de Valldemossa km 7.5, 07122 Palma de Mallorca (Baleares), Spain.

***Corresponding author:**

Pharmaceutical Analytical Chemistry Department, Faculty of Pharmacy, Assiut University, Assiut 71526, Egypt. Tel: +2-01144438525

E-mail: [noha.hosni@aun.edu.eg](mailto:noha.hosni@aun.edu.eg)

ORCID ID: [0000-0002-6176-7702](https://orcid.org/0000-0002-6176-7702)

**Figure 1S.** The effect of **(A)** EUC volume, **(C)** number of cycles of electro-polymerization of EUC, and (**E**) the effect of phosphate buffer pH on the oxidation current intensities of 5-FU (15 μM) and EMPA (7.5 μM). (**B, D, and F**) the corresponding voltammograms.

**Figure 2S.** The effect of **(A)** XO volume, **(C)** number of cycles of electro-polymerization of XO, and **(E)** the effect of phosphate buffer pH on the oxidation current intensities of 5-FU (15 μM) and EMPA (7.5 μM). **(B, D, and F)** the corresponding voltammograms.

**Figure 3S**. The effect of different scan rates (0.05–0.9 V s^-1^) on of 1.05×10^-4^ M, 5.3×10^-5^ M of 5-FU and EMPA, respectively in Britton-Robinson buffer solution (0.04 M, pH 8.0) employing poly-XO@poly-EUC/PGE sensor. Panel **(A)**: is the plot of oxidation peak currents (Ip) of 5-FU and EMPA versus scan rate (ν), **(B)** is the plot of logarithms of anodic peak currents (log Ip) versus logarithms of scan rate (log ν), **(C)** is the plot of oxidation peak current (Ip) of 5-FU and EMPA versus square root of scan rate (ν^½^), and **(D)** is the plot the plot of peak potentials (Ep) versus logarithms of scan rate (log ν).

**Figure 4S.** The SW voltammogram for 5-FU (15 μM) and EMPA (7.5 μM) employing poly-XO@poly-EUC/PGE sensor using different types of buffers at pH 8.0.

**Figure 5S.** The effect of **(A)** step size, **(C)** pulse size, and **(E)** frequency on the oxidation current intensities of 5-FU (15 μM) and EMPA (7.5 μM). **(B, D, and F)** the corresponding SW voltammogram for each effect.

**Figure 6S.** Bar graphs represent **(A)** the reproducibility and **(B)** stability of the modified poly-XO@poly-EUC/PGE probe.

**Table 1S.** Robustness of the established SWV method.

| **Parameter** | **5-FU** | | **EMPA** | |
| --- | --- | --- | --- | --- |
|  | **% Recovery ± SD^a^** | **RSD %** | **% Recovery ± SD^a^** | **RSD %** |
| **pH of Britton-Robinson buffer (8.0)** | | | | |
| 7.9 | 97.22 ± 1.08 | 1.11 | 97.83 ± 1.28 | 1.31 |
| 8.1 | 98.18 ± 1.13 | 1.16 | 102.51 ± 0.24 | 0.24 |
| **Step size (7 mV)** | | | | |
| 6 mV | 99.04 ± 0.79 | 0.80 | 101.86 ± 0.16 | 0.16 |
| 8 mV | 100.39 ± 1.14 | 1.14 | 100.08 ± 0.84 | 0.84 |
| **Pulse size (10 mV)** | | | | |
| 9 mV | 97.47 ± 1.11 | 1.14 | 97.50 ± 1.10 | 1.12 |
| 11 mV | 102.56 ± 0.32 | 0.31 | 99.60 ± 0.35 | 0.35 |
| **Frequency (12 Hz)** | | | | |
| 11 Hz | 99.78 ± 0.86 | 0.86 | 101.88 ± 0.23 | 0.23 |
| 13 Hz | 100.57 ± 0.50 | 0.49 | 103.80 ± 0.97 | 0.94 |

^a^ Average of three measurements. Values at the optimum conditions were written in parenthesis.

**Table 2S.** Selectivity of the established SWV method.

| **Interfering material** | **5-FU** | | **EMPA** | |
| --- | --- | --- | --- | --- |
|  | **% Recovery ± SD^a^** | **RSD%** | **% Recovery ± SD^a^** | **RSD%** |
| Sodium chloride | 99.49 ± 0.88 | 0.88 | 98.55 ± 0.34 | 0.34 |
| Magnesium chloride | 101.27 ± 1.10 | 1.09 | 101.05 ± 0.91 | 0.90 |
| L-Ascorbic acid | 99.30 ± 1.18 | 1.18 | 102.06 ± 1.30 | 1.27 |
| Citric acid | 98.67 ± 0.93 | 0.94 | 97.55 ± 0.17 | 0.17 |
| Uric acid | 100.04 ± 0.44 | 0.44 | 97.44 ± 1.30 | 1.34 |
| Starch | 96.38 ± 1.00 | 1.04 | 98.40 ± 0.39 | 0.39 |
| Ondansetron | 100.23 ± 0.40 | 0.40 | 100.87 ± 1.22 | 1.21 |
| Sitagliptin | 98.75 ± 0.30 | 0.31 | 98.26 ± 1.39 | 1.42 |
| Pioglitazone | 97.42 ± 0.51 | 0.52 | 102.37 ± 2.10 | 2.05 |
| Dexamethasone | 100.43 ± 0.37 | 0.37 | 101.51 ± 0.31 | 0.31 |
| Metformin | 100.91 ± 1.58 | 1.56 | 99.86 ± 0.92 | 0.92 |

^a^ Average of three determinations. Concentrations of 5-FU, EMPA and interferent were 15, 7.5, and 150 μM, respectively.

**Table 3S.** Comparison between the established SWV method and other reported methods for the analysis of the target drugs.

| **Ref.** | **Application** | **LOD**  **(µM)** | **Linearity range**  **(µM)** | **Drugs** | **Technique** |
| --- | --- | --- | --- | --- | --- |
| **This work** | Bulk forms and spiked human plasma | ‎0.330  0.020 | ‎1.5-25‎,  32.5-100.0  ‎  0.25-15.0‎,  ‎16.25-50.0‎ | **5-FU**  **EMPA** | **SWV** |
| [22] | Forced degradation studies  Dried blood spots | 0.330 | 7.69-1153 | **5-FU** | **RP-HPLC** |
| [81] |  | ----- | 0.769–7.692 |  |  |
| [27]  [26] | Pure and tablet formulation  Rat plasma and pharmacokinetic study | 0.350 | 11.11–55.56 | **EMPA** |  |
|  |  | 0.056 | 0.056-2.189 |  |  |
| [82] | Pure and dosage form | 0.144 | 0.444—3.333 | **EMPA** | **Spectrophotometry** |
| [17] | Pure and tablet | ------ | 22.22–111.11 |  |  |
| [15] | Pure and dosage form | 2.99 | 13.00–78.05 | **5-FU** | **Spectrofluorimetry** |
| [83]  [84]  [19] | Dosage form and Spiked human plasma  Dosage form and Spiked human plasma  Spiked rabbit plasma and pharmacokinetic study | 6.26  5.36  0.67 | 22.2-488.89  17.78-533.33  2.22-133.33 | **EMPA** |  |
|  |  |  |  |  |  |
| [73] | Dosage form | 10.7 | 90.0 -300 | **5-FU** | **SWV** |
| [33] | Pharmaceutical formulations and biological fluids | 1.71 | 7.94–107 | **EMPA** |  |
| [28]  [30] | Pure form  Pure form | 2.10  3.41 | 10.0-70.0  20.0-80.0 | **5-FU** | **DPV** |
| [35]  [36] | Spiked human plasma  Spiked human urine | 4.15 ‎  ------ | 10.0–70.0  10.0–70.0 | **EMPA** |  |
| [20]  [79]  [24]  [80] | Spiked rabbit plasma and pharmacokinetic study | ------ | 11.259–116.471 | **5-FU** | **HPTLC** |
|  | Stability indicating assay  Tablets and spiked rabbit serum  Tablets and spiked human plasma | 0.499  16.8  1.76 | 22.22-155.56  133.33-1333.33  5.56-1111.11 | **EMPA** |  |
|  |  |  |  |  |  |
